# Effect of berberine on LPS-induced intestinal epithelial injury and m^6^A methylation in broilers

**DOI:** 10.1016/j.psj.2025.105677

**Published:** 2025-08-12

**Authors:** Lin Yuan, Wanli Li, Guoxi Li, Wei Jin, Bingxun Wang, Shengli Li, Haoyu Wang

**Affiliations:** a Key Laboratory of Livestock and Poultry Breeding and Nutrition Regulation in Henan Province, Institute of Animal Husbandry, Henan Academy of Agricultural Sciences, Zhengzhou 450002, China; b The Shennong Laboratory, Zhengzhou Henan 450002, China; c College of Animal Science and Technology, Henan Agricultural University, Zhengzhou 450046, China

**Keywords:** Broiler, Berberine, Intestinal epithelium injury, Intestinal inflammation, Methylation

## Abstract

This study sought to explore the effects of and potential mechanisms underlying berberine (BBR) in lipopolysaccharide (LPS)-induced intestinal epithelium injury in broilers. Ninety 19-day-old male AA broilers were randomly divided into five groups: the negative control group (NC), positive control group (PC), and three treatment groups (T1, T2, and T3). On the 21st day of age, the control group was orally administered saline, the LPS-challenged group was orally administered LPS (3 mg/kg body weight), and the treatment groups were orally administered BBR (6, 12, 18 mg/kg body weight) along with LPS (3 mg/kg body weight). Afterwards, in vitro cell experiments were used for validation. The results revealed that the oral administration of LPS caused intestinal inflammation and cell apoptosis and disrupted the intestinal barrier and function. However, simultaneous administration of high-dose BBR alleviated LPS-induced adverse changes in intestinal morphology and mucosal barrier integrity by decreasing the expression of inflammatory cytokines and apoptotic proteins, increasing the expression of tight junction proteins, and inhibiting LPS-induced increases in m^6^A methylation and METTL3 expression. Our findings indicated that BBR addition attenuated LPS-induced intestinal epithelium injury by increasing the expression of tight junction proteins and increasing the anti-inflammatory and antiapoptotic capacity of broiler chickens, possibly via coregulating METTL3 protein expression and m^6^A methylation levels. The research will provide theoretical support for the application of natural alkaloid anti-inflammatory additives in poultry farming.

## Introduction

During intensive poultry farming, broilers are often infected by various pathogens, which trigger immune responses that result in damage to the intestinal barrier and intestinal inflammation, seriously endangering the intestinal health of the animals (Szkopek et al., 2024). Lipopolysaccharide (LPS), a component of the cell wall of gram-negative bacteria, is one of the main factors causing intestinal mucosal damage, LPS is released when the gut microbiota is imbalanced or the barrier function is impaired, which can activate the immune pathway through the destruction of tight junction proteins and trigger intestinal inflammatory reactions, ultimately affecting the growth performance and health status of poultry (Liu et al., 2023). LPS stimulation not only induces intestinal inflammation but also increases intestinal permeability and leads to a decrease in the expression levels of intestinal barrier function-related proteins, such as claudins, zona occludens (ZOs), and occludins (Zhang et al., 2024). Therefore, LPS is commonly used to establish models of intestinal damage and immune stress in broilers (Lv et al., 2020). Berberine (BBR) is an isoquinoline alkaloid isolated from the traditional Chinese medicine Coptis chinensis. It has various clinical effects, including antidiarrheal, antibacterial, anti-inflammatory, and antitumor properties (Hesari et al., 2018; Yarla et al., 2016). It can be used to treat digestive system diseases, such as peptic ulcers, gastritis, and enteritis (Song et al., 2020), by inhibiting bacteria and toxins, suppressing secretion and peristalsis, and protecting the intestinal epithelial barrier. Studies have shown that m^6^A methylation may be a potential mechanism underlying the anti-inflammatory (Shen et al., 2020), anticancer (Zhao et al., 2021), antioxidative stress (Zhang et al., 2022a), and damage repair (Hu et al., 2024) effects of BBR.

N6-methyladenosine (m^6^A) is the most prevalent internal modification in eukaryotic mRNAs. Its modification is regulated primarily by three types of m^6^A regulatory proteins: "writers" (methyltransferases), "erasers" (demethylases), and "readers" (m^6^A recognition proteins) (Shi et al., 2019). m^6^A is involved in almost all processes of mRNA metabolism, including RNA transcription, translation, and degradation. It alters the expression of target genes, thereby affecting corresponding cellular processes and physiological functions (Roundtree et al., 2017). Studies have shown a close relationship between m^6^A modification and inflammatory responses (Xu et al., 2022). m^6^A plays an important role in cisplatin induced AKI and BBR may alleviate this process (Shen et al., 2020). Studies in zebrafish-hepatocyte have also shown that BBR could regulate cellular oxidative stress, apoptosis and autophagy by mediating Camk1db m^6^A methylation (Zhang et al., 2022b). BBR could also blocked breast cancer cell growth and metastasis partly by regulating METTL3-mediated m6A modification of FGF7 Mrna (Fu et al., 2024).

Currently, there is limited research on whether m^6^A modification is involved in regulating the alleviation of LPS-induced intestinal damage in broilers through BBR. Therefore, the objectives of this study were to further investigate the effects of BBR on LPS-induced intestinal damage in broilers and the levels of m^6^A regulatory proteins and to elucidate the mechanisms by which BBR alleviates intestinal damage in broilers affected by LPS. The research will provide theoretical support for the application of natural alkaloid anti-inflammatory additives in poultry farming, and help maintain the intestinal health of chickens, improve feed conversion rate, and reduce the use of antibiotics in farming. This will respond to the global policy trend of "reducing and replacing antibiotics" and promote the sustainable development of poultry farming.

## Materials and methods

### Animal ethics statement

The experimental methods were performed in accordance with the guidelines of the Good Experimental Practices, adopted by the Institute of Animal Husbandry. Additionally, All animal procedures in this study complied with the Institutional Animal Care and Use Committee (IACUC) guidelines, authorized by the Henan Academy of Agricultural Sciences’ Institute of Animal Husbandry (Zhengzhou, China, ID: 2024-5 [2]).

### Animals, treatments, and feeding management

Ninety 19-day-old male AA broilers, weighing approximately 700 grams each, were randomly divided into five groups, the negative control group (NC), positive control group (PC), and three treatment groups (T1, T2, and T3), with 3 cages per group and 6 broilers per cage. On the 21st day of age, the control group was orally administered saline, the LPS-challenged group was orally administered LPS (3 mg/kg body weight), and the treatment groups were orally administered BBR (6, 12, 18 mg/kg body weight) along with LPS (3 mg/kg body weight). The administration was carried out by mixing LPS or BBR with saline and administering it to each broiler using a syringe. The control and LPS-treated groups received the same volume of saline. The experimental broilers were housed in wire mesh cages in an environmentally controlled room with continuous lighting. Throughout the experiment, the broilers had free access to a standard corn–soybean meal-based diet. The composition and nutritional levels of the basal diet (Table 1) met the standards set by the Chinese ministry of Agriculture in 2004. All the diets were fed in powder form.

**Table 1:** Diet compositions and nutrient levels.

Parameter	Basal diet
Ingredients, %	
Corn	62.13
Soybean meal	30.32
Vegetable oil	3.97
Dicalcium phosphate	1.45
Limestone	1.35
Table salt	0.30
*DL*-Methionine	0.15
Mineral premix	0.10
*L*-Lysine	0.10
Choline chloride	0.10
Vitamin premix	0.03
Nutrient levels	
ME, kcal/kg	3084.20
Crude protein	20.07
Met, %	0.41
Lys, %	1.03
Calcium, %	0.91
Non-phytate phosphorus, %	0.41

### Sample collection

At the end of 28 days, one broiler was randomly selected from each cage for sampling. Blood samples were extracted from the pterygoid vein, placed into sterile blood collection vessels containing heparin sodium anticoagulant, and centrifuged at 3000 × g at 4 °C for 15 minutes. The serum was stored at -20 °C. After blood samples were collected, the broilers were anesthetized by intravenous injection of 25 mg/kg of thiopental sodium (Takara, Dalian, China), after a few minutes, the broilers fell, general weakness, reaction disappeared, and then euthanized by carotid artery bloodletting. The ileum was collected and rinsed with 9 % normal saline several times. One part was preserved and fixed in 4 % paraformaldehyde solution for histopathological analysis, and the other part was placed in a 2 ml RNase-free centrifuge tube. After quick freezing in liquid nitrogen, the samples were stored at -80 °C for gene and protein expression analysis.

### Histopathological analysis

The fixed ileum samples were trimmed, dehydrated, embedded, sectioned, stained, and mounted. The morphology of each section was observed under an upright bright-field microscope (Nikon Eclipse Ci-L, Japan). For each sample, 3 well-oriented villus height (VH) and crypt depth (CD) were measured, and the VH/CD ratio was calculated.

### Measurement of serum inflammatory cytokine concentrations

The serum levels of tumor necrosis factor-α (TNF-α), interleukin-1 (IL-1β) and interleukin-6 (IL-6) in broilers were determined with an ELISA kit from Shanghai Coibo Co., Ltd. (Coibo, Shanghai) following the manufacturer’s instructions.

### Polymerase chain reaction (PCR)

Total RNA was extracted from the ileum using TRIzol reagent (Invitrogen, Carlsbad, CA). The purity and concentration of total RNA were determined via an ultraviolet spectrophotometer (NanoDrop 2000c, Thermo Scientific, USA). The cDNA was reverse-transcribed using the swescript RT I first strand cDNA synthesis kit (with gDNA remover) (Servicebio, Wuhan). Quantitative real-time PCR was performed via the use of articanatm SYBR qPCR mix on an ABI quantstudio6 Felx real-time system (ABI, USA). The reaction mixture (10 μL) contained 5 μL of ArtiCanATM SYBR qPCR mix, 0.2 μL of forward and reverse primers (10 μmol/L), 0.2 μL of 50 × Rox reference dye I, 1 μL of template and 3.4 μL of ddH2O. The PCR cycle conditions were as follows: 95 °C for 60 s, followed by 40 cycles of 95 °C for 10 s and 60 °C for 30 s. The specificity of the PCR products was evaluated by melting curves. Table 2 lists the primer sequences for the target genes and reference genes. The relative mRNA expression levels of the target genes were calculated via the 2^−ΔΔCT^ method, and the housekeeping gene GAPDH was used as an internal control.

**Table 2:** Sequences of the primers used for quantitative real-time PCR.

Gene name	Sequence (5′∼3′)	Product size, bp	Accession number
IL-6	F-ATAAATCCCGATGAAGTGGTC	143	NM_204628.2
	R-CACGGTCTTCTCCATAAACG		
IL-1β	F-TGCTGGTTTCCATCTCGTAT	127	NM_204524.2
	R-ACGGGACGGTAATGAAACA		
TNF-α	F-CCCTACCCTGTCCCACAA	147	NM_204267.2
	R-GGCGGTCATAGAACAGCAC		
claudin-1	F-GGATGGGTATCATCATCAGCAC	217	NM_001013611
	R-GGAGTATGGCGGCCACCATC		
occludin	F-AGCTACAGCTACGGCTACGG	205	XM_025144247
	R-GAGCATGACGAAGGCCAGC		
METTL3	F-TCCCACCATCGTCACCTACG	188	XM_040655036.2
	R-ACGCCTGCTTACGGGATTTC		
METTL14	F-TTGGCTCAGCAGTTAGGAGC	87	NM_001031148.2
	R-TGTTTCAGCAATCTCCCGCT		
FTO	F-TACTGCTAGTTCTTGCCGAGC	76	NM_001185147.1
	R-CTCTCCGCTGCTTCTGACTG		
GAPDH	F-GGACCAGGTTGTCTCCTGTG	151	XM_049824763.1
	R-TCCTTGGATGCCATGTGGAC		

### m6A methylation

m^6^A dot blotting was used to detect the methylation level of total RNA in the cells. The total RNA was denatured at 65 °C for 5 min and immediately placed on ice. A 2 µl RNA sample was added to a nylon membrane (Biyuntian, Shanghai) and subjected to UV crosslinking for 10 min. Tbst was used to wash the membrane for 5 min to remove the unbound RNA. The tbst was discarded, and a sealing solution (Biyuntian, Shanghai) was added for 1 h of blocking. The sample was incubated with a specific m^6^A antibody (Sanying, Wuhan) at 4°C overnight. The Tbst was washed 4 times for 10 min each time. The secondary antibody (Sanying, Wuhan) was then added, and the mixture was incubated for 1 h. The Tbst was washed 4 times for 10 min each time. ECL (Biyuntian, Shanghai) substrate color rendering was performed, and photos were taken. After photographing, the membrane was placed in 10 ml of methylene blue staining buffer and incubated at room temperature for 30 min. The background was cleaned with ultrapure water, and photographs were taken with white light.

### Immunohistochemistry (IHC)

The levels of the ileal barrier-associated protein claudin-1 and methylation-associated enzymes were determined via immunohistochemistry (IHC). In brief, paraffin-embedded tissue chips were dried at 90°C for 4 h, dewaxed in xylene, and then rehydrated in a series of graded ethanol solutions. EDTA solution (1 mM, pH 9.0) was used for antigen retrieval. A 0.3 % hydrogen peroxide solution was used to block endogenous peroxidase activity in the tissue sections, which was followed by rinsing with PBS for 5 min and blocking with 3 % BSA solution. The sections were then incubated with primary antibodies (Sanying, claudin-1: 13050-1-AP, 1:3000; METTL3: 15073-1-AP, 1:2000; METTL14: 26158-1-AP; ZO-1: 21773-1-AP, 1:5000) at 4°C overnight, followed by incubation with an HRP-labeled secondary antibody at 37°C for 30 min. Diaminobenzene was used as the chromogen, and hematoxylin was used as the nuclear counterstain. The sections were then dehydrated, cleared, and mounted.

### Western blot

Western blotting (WB) was used to evaluate the protein expression of the intestinal barrier-associated methylase METTL3 in the chicken ileum. First, a polyacrylamide gel was prepared, and electrophoresis was performed after the required samples were added. Next, after the target strip was cut and the membrane was rotated, the PVDF membrane was soaked in tbst (sealing solution) containing 5 % skim milk powder, shaken at room temperature for 2 h, washed three times with PBS, and incubated with a primary antibody (Sanying, ZO-1: 21773-1-AP; Caspase-3: 25128-1-AP, 1:1000; β-actin: 66009-1-Ig, 1:10000) overnight. On the second day, the PVDF membrane was fully washed 4 times for 10 min per wash and then incubated with the secondary antibody for 2 hours. After the samples were washed 4 times again, color was developed with an enhanced chemiluminescence (ECL) kit, and the samples were placed in a chemiluminescence instrument for development and photography.

### Cells

DF-1 chicken fibroblasts (ATCC CRL-12203), which were maintained in our laboratory, were cultured in Dulbecco’s modified Eagle’s medium (DMEM; Servicebio, Wuhan) with 10 % foetal bovine serum (FBS; Servicebio) at 37°C and 5 % CO_2_. The pre experiment for concentration screening of LPS and BBR is shown in Appendix supplementary data 2.

## Results

### Effects of LPS and BBR on the histopathology of the ileum in broilers

**Fig. 1:**
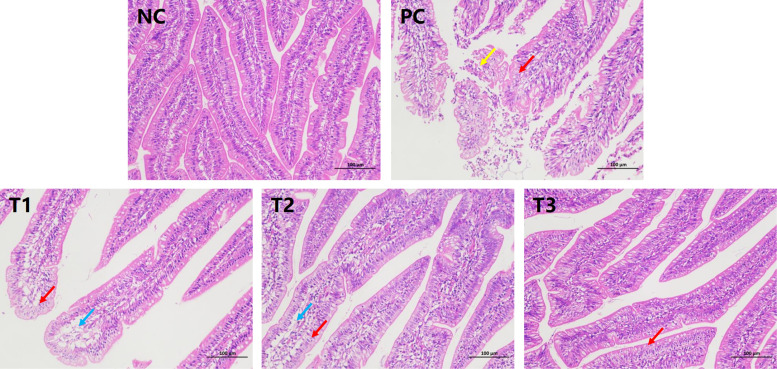
Histopathological features observed in the ileum of 28-day-old chickens orally administered LPS and berberin.

**Fig. 2:**
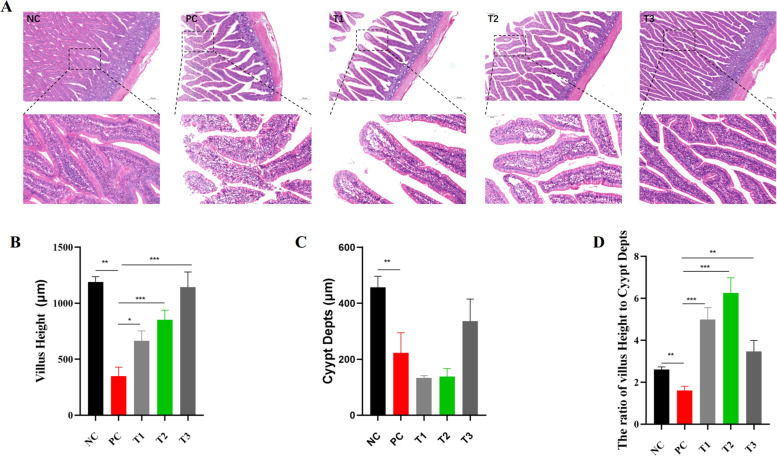
Changes in villus height (VH) and crypt depth (CD) in 28-day-old chickens after the oral administration of LPS and BBR.

A histopathological micrograph of the intestine is shown in Fig. 1, Fig. 2A-D and Appendix supplementary data 1. Pathological examination revealed that the ileum and intestinal villi were intact in the NC group. Compared with those in the NC group, all samples from the ileum in the PC group presented watery degeneration of intestinal villus epithelial cells, some samples from the intestinal villus epithelial cells fell off. The VH, CD and VH/CD significantly decreased in the PC group (Fig. 2B-D).

Compared with those in the PC group, in the T1 ileum group, the top of the intestinal villi epithelium was separated from the lamina propria in some samples, and the number of goblet cells in individual samples was reduced. In the T2 ileum group, the number of exfoliated samples from the intestinal villus epithelial cells decreased, the intestinal villus epithelium of individual samples was separated from the lamina propria. In the T3 ileum group, the number of exfoliated samples from the intestinal villus epithelial cells decreased. The VH, CD and VH/CD significantly increased in the T1, T2 and T3 groups (Fig. 2B-D).

### Effects of LPS and BBR on proinflammatory cytokines in broilers

**Fig. 3:**
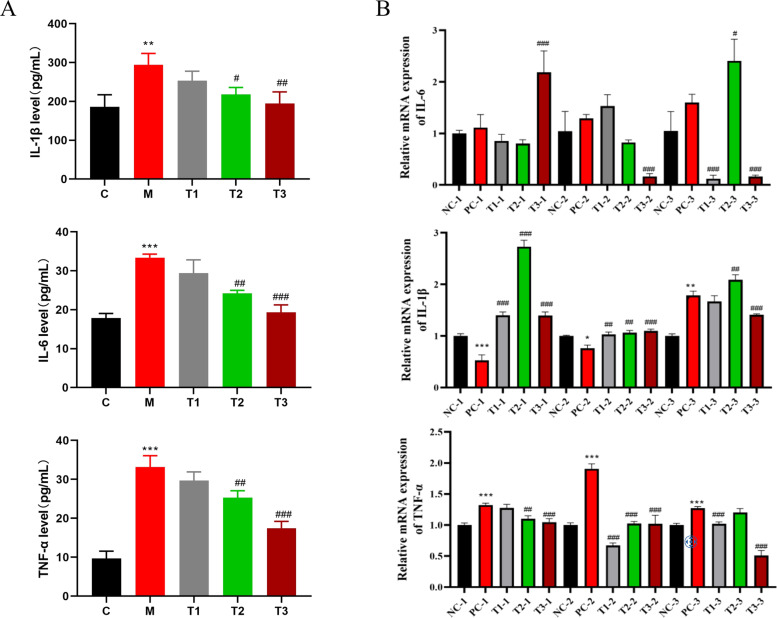
Changes in proinflammatory cytokines in 28-day-old chickens after the oral administration of LPS and BBR.

The levels of proinflammatory cytokines in broiler serum are shown in Fig. 3A. The serum levels of IL-1β, IL-6 and TNF-α in the LPS group were significantly greater than those in the NC group, whereas the serum levels of proinflammatory cytokines in the T2 and T3 groups were significantly lower than those in the LPS group. The symbols *, **, *** indicate significant differences between the PC group and the NC group at the 0.05 (*P* < 0.05), 0.01(*P* < 0.01), and 0.001 (*P* < 0.001) levels, respectively. The symbols #, ##, ### indicate significant differences between the T1, T2, T3 groups and the PC group at the 0.05 (*P* < 0.05), 0.01(*P* < 0.01), and 0.001 (*P* < 0.001) levels, respectively, and the same below.

**Fig. 4:**
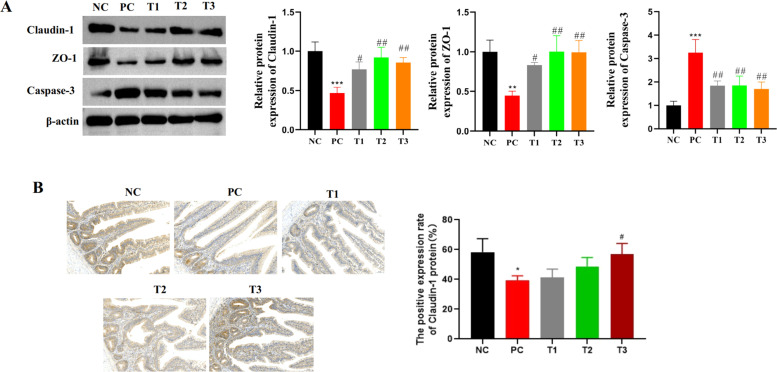
Changes in the levels of ileal barrier-associated proteins in 28-day-old chickens after the oral administration of LPS and BBR.

The mRNA expression levels of the ileal proinflammatory cytokines are shown in Fig. 3B. Across three biological replicates, the expression level of IL-6 mRNA in the PC group tended to increase, but there was no significant difference compared with that in the NC group. Compared with that in the PC group, the expression of IL-6 mRNA in the T group decreased with increasing drug concentration. The IL-1β mRNA results of the first and second biological replicates revealed that the expression level of IL-1β mRNA in the PC group was significantly lower than that in the NC group. Compared with that in the PC group, IL-1β mRNA expression in the T1 group was significantly increased. However, in the third biological replicate, the expression of IL-1β mRNA in the PC group was greater than that in the NC group. Moreover, with increasing drug concentration, the expression of IL-1β mRNA in the T group was significantly lower than that in the PC group. Compared with that in the NC group, the TNF-α mRNA level in the PC group was significantly greater, and compared with that in the PC group, the expression of TNF-α mRNA in the T1, T2 and T3 groups decreased gradually. (Fig. 4)

### Effects of LPS and BBR on ileal barrier-associated proteins in broilers

WB was used to detect the expression levels of claudin-1, ZO-1 and caspase 3 in intestinal tissue. The results revealed that the protein expression level of caspase-3 in the PC group was significantly greater than that in the NC group, and the protein expression levels of claudin-1 and ZO-1 were significantly lower. Compared with those in the PC group, the protein expression levels of caspase-3 in the T1, T2 and T3 groups were lower, whereas the protein expression levels of claudin-1 and ZO-1 were greater, and the changes in the T3 group were more significant.

Detection of claudin-1 protein expression by IHC revealed that the positive expression rate of claudin-1 protein in the PC group was significantly lower than that in the NC group. Moreover, the positive expression rate of claudin-1 protein in the T3 group was significantly greater than that in the PC group. The results revealed that the expression of claudin-1 was significantly decreased during the development of broiler enteritis and that claudin-1 expression was restored by high-concentration BBR treatment.

### Effects of LPS and BBR on m6A methylation-modified mRNA levels in broilers

**Fig. 5:**
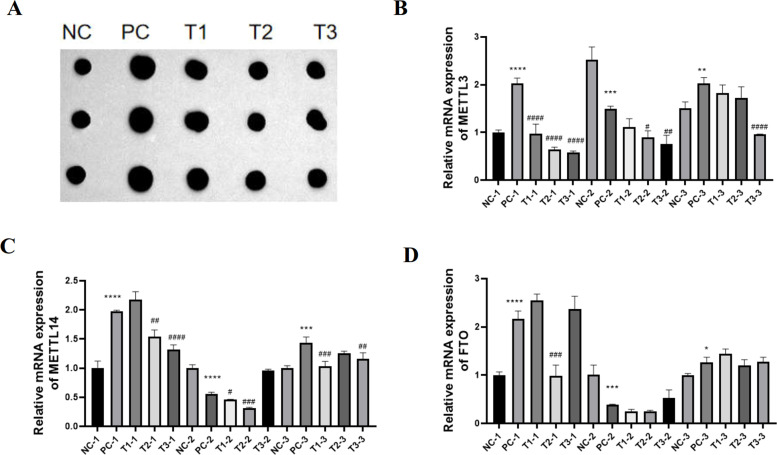
Changes in m6A methylation levels in 28-day-old chickens after the oral administration of LPS and BBR.

The m^6^A methylation level detected by the m^6^A dot blot test is shown in Fig. 5A. Compared with that in the NC group, the level of m^6^A-modified RNA in the PC group was greater, and compared with that in the PC group, the level of m^6^A-modified RNA in the T1, T2, and T3 groups was lower. These results suggest that m^6^A modification may be involved in model construction and treatment.

The qPCR results revealed no obvious change in FTO mRNA. METTL3 and METTL14 mRNAs were regularly expressed in the first and third biological replicates; that is, the model group (PC) was highly expressed, and the expression level decreased after treatment in a dose-dependent manner.

### Effects of LPS and BBR on m6A methylase protein levels in broilers

**Fig. 6:**
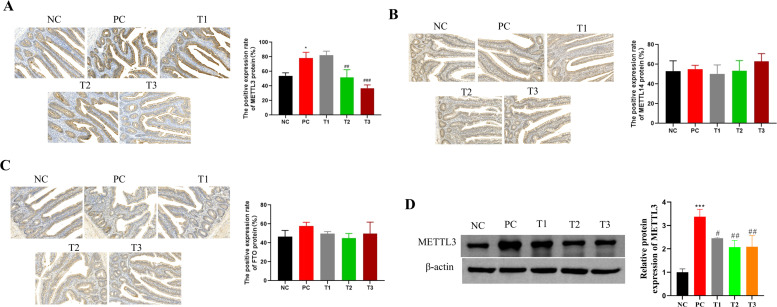
Changes in m6A methylase protein levels in 28-day-old chickens after the oral administration of LPS and BBR.

The protein expression levels of the methylases METTL3, METTL14 and FTO detected by IHC are shown in Fig. 6A‒C. Compared with that in the NC group, the percentage of METTL3 protein-positive cells in the PC group was significantly greater. Compared with that in the PC group, the percentage of METTL3 protein-positive cells in the T2 and T3 groups was significantly lower. The results revealed that METTL3 protein expression increased abnormally during the process of broiler enteritis and that METTL3 protein expression was restored after BBR treatment in a dose-dependent manner. FTO and METTL14 did not significantly differ among the groups.

Considering that the qPCR and IHC results revealed that METTL3 was highly expressed in the PC group and that its expression decreased after treatment, we further used WB to detect the protein expression level of METTL3. The results revealed that the protein expression level of METTL3 in the PC group was significantly greater than that in the NC group. Compared with that in the PC group, the protein expression level of METTL3 in the T1, T2 and T3 groups was lower.

### In vitro cell validation experiment

**Fig. 7:**
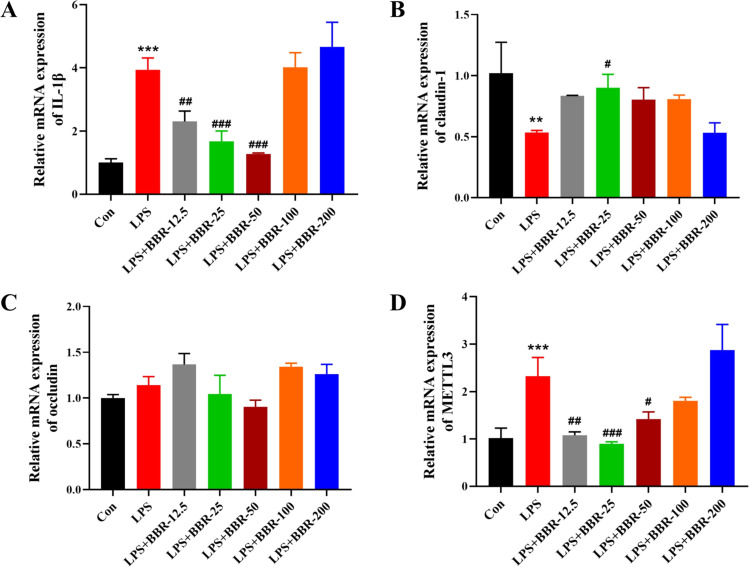
Changes in IL-1β, claudin-1, occludin and METTL3 mRNA levels in DF-1 chicken fibroblasts after stimulated of LPS and BBR.

The mRNA expression levels of the IL-1β, claudin-1, occludin and METTL3 in DF-1 cells are shown in Fig. 7A‒D. Compared with the NC group, the LPS group showed a significant increase in IL-1β and METTL3 mRNA levels, a significant decrease in claudin-1 mRNA levels, and no significant difference in occludin mRNA levels.

Compared with the LPS group, low concentrations of BBR significantly reduced the levels of IL-1β and METTL3 mRNA, 50 μM BBR can significantly reduce the level of claudin-1 mRNA, BBR stimulation had no significant effect on occludin mRNA levels.

## Discussion

As the initial protective barrier against harmful substances, the intestinal barrier plays an important role in maintaining the stability of the intestinal environment and preventing the invasion of harmful substances (Beumer and Clevers, 2021). Maintaining integrity of the intestinal epithelial morphology is essential for maintaining intestinal barrier function (Huang et al., 2022b). This study revealed that LPS injection caused clear pathological changes in the ileum of broilers and that the intestinal villi exhibited morphological damage and inflammation. These findings indicate that this model effectively leads to impaired intestinal function, which is consistent with the previous findings of Liu et al. (Liu et al., 2023, Wang et al., 2024c). The results of this study revealed that, compared with those of LPS-challenged broilers, the intestinal villus epithelial cells of BBR-treated broilers shed fewer samples, and the crypt depth of some samples increased. These results suggest that BBR can protect the structure of intestinal villus epithelial cells from LPS damage. A study on weaned piglets infected with enterotoxic *Escherichia coli* revealed that BBR can increase the height of jejunal villi, the mucosal thickness, and the number of goblet cells in villi and crypts (Du et al., 2024). BBR can also increase the number of goblet cells and villus length in the intestinal tract in a rat model of diabetes (Wang et al., 2021). BBR has been widely used in animal production, it could effectively alleviated fatty liver hemorrhagic syndrome induced by high-energy and low-protein by reshaping the microbial and metabolic homeostasis within the liver-gut axis (Cheng et al., 2024). BBR was also found to exhibit anticoccidial activity, berberine induces hosts to exert anti-Eimeria activity by attenuating the inflammatory and oxidative stress response, by impairing apoptotic processes, and by activating local innate immune responses and epigenetic mechanisms in the host jejunum (Dkhil et al., 2015). BBR has a good therapeutic effect on Salmonella infection in chicks, as it inhibits the occurrence and development of Salmonella-induced intestinal inflammation by regulating the balance of the gut microbiota and the expression of related genes (Li Yang et al., 2024). But there were also studies found that BBR supplementation improves chicken gut morphology toward decreased inflammation, which is likely not mediated by the induced gut microbiota shifts (Dehau et al., 2023). However, the epigenetic modification regulation mechanism of BBR in the body has not been elucidated, and its safety risks are not yet clear.

LPS is an endotoxin that exerts its effects via cell-surface TLR4, inducing the release of proinflammatory factors (Chae, 2018). The inflammatory factors TNF-α, IL-1β and IL-6 are the main cytokines that mediate the early inflammatory response and play important roles in immune regulation by binding to cell-surface receptors on immune cells (Ding et al., 2023). In this study, LPS administration increased the levels of the inflammatory cytokines TNF-α, IL-6 and IL-1β in the serum and the relative expression of TNF-α in the ileum of broilers. After BBR treatment, the concentrations of proinflammatory cytokines in the serum of broilers in the T2 and T3 groups were significantly lower than those in the LPS group, indicating that BBR reduces the inflammatory response. Previous studies have shown that taking BBR can significantly alleviate the increase in serum IL-6, IL-1β, and TNF-α caused by *Escherichia coli* infection in chicks (Meng et al., 2024) and alleviate the increase in IL-6, IL-8, and TNF-α expression in the intestine caused by *Escherichia coli* infection in weaned piglets (Du et al., 2024). Studies in mice have also shown that BBR can improve the clinical symptoms of various types of arthritis by downregulating the expression levels of inflammatory factors, such as IL-1β, IL-6, and TNF-α (Li et al., 2023).

The intestinal barrier is not only key for the body to resist harmful pathogens from the outside but is also the basis for maintaining intestinal mucosal epithelial permeability and barrier function (Liu et al., 2023). Our results indicate that LPS treatment significantly reduced the levels of claudin-1 and ZO-1 and increased the expression of caspase-3. Similar results were obtained when LPS was used to induce intestinal inflammation in chicken embryos (Yu et al., 2022). claudin-1 is the major cytoplasmic transmembrane protein, whereas ZO-1 is the most important cytoplasmic adaptor protein (Varasteh et al., 2015). They are both major functional proteins that participate in constructing tight intercellular junctions between cells, serving as the most powerful means to maintain the intestinal mucosal barrier and strictly ensure the integrity and normal abilities of tight intercellular junctions (Chen et al., 2020). Caspase-3 plays an executive role in apoptosis, indicating that LPS can induce intestinal inflammation and apoptosis (Shen et al., 2021; Zhang et al., 2022c). The results of this study showed that the addition of BBR can improve the decrease in claudin-1 and ZO-1 and the increase in Caspase-3 caused by LPS. A study in piglets also revealed that BBR increased the expression of ZO-1, ZO-2, claudin-1, and Occludin in the ileum; reduced intestinal epithelial cell apoptosis; and decreased the expression of CASP3 and CASP9 in the duodenum and ileum (Du et al., 2024). Previous work has shown that elevated peripheral mucosal claudin-1 and ZO-1 levels contribute to the regulation of intestinal integrity and barrier function (Zhang et al., 2017). These results suggest that BBR could improve intestinal mucosal barrier function by enhancing the antiapoptotic ability of intestinal cells.

In recent years, there has been a surge in research emphasizing the pivotal role of m^6^A methylation modification in intestinal diseases (Wang et al., 2024b, Zhang et al., 2023). In adult mice, m^6^A knockout in the intestinal epithelium results in symptoms such as weight loss, shortened colon length, diarrhea, and severe colitis, revealing that m^6^A affects the development and homeostasis of the colon epithelium by regulating the function of intestinal stem cells (Zhang et al., 2022b). The loss of m^6^A methylation in macrophages aggravates cytokine production and myocardial damage in mice under septic conditions (Wang et al., 2023). Research has shown that, after infection with *Cryptosporidium*, the methylation status of m^6^A in intestinal epithelial cells changes, affecting the expression of immune-related genes and thus affecting the intrinsic defense of the intestinal epithelium (Xia et al., 2021). Wu et al. (2022) investigated the potential role of m^6^A and its regulatory factors in mediating the susceptibility of pig small intestinal epithelial cells to *Escherichia coli* F18, which could contribute to the development of new methods for preventing bacterial diarrhea in piglets (Wu et al., 2022). These findings suggest that m^6^A plays a certain role in maintaining the intestinal mucosal barrier against pathogen infections.

METTL3 and METTL14, as key enzymatic writers of m^6^A, catalyze m^6^A modification in RNA and play important roles in regulating intestinal homeostasis (Jiang et al., 2021). FTO, a m^6^A demethylase, plays pivotal roles in intestinal injury by inflammation (Wang et al., 2025). Our study revealed that LPS challenge increased m^6^A mRNA and METTL3 protein levels in the chicken ileum but had no significant effect on METTL14 or FTO protein levels. However, another study revealed that, after LPS challenge, the level of METTL14 in macrophages increases, whereas the level of FTO protein decreases, increasing the level of m^6^A (Wang et al., 2023). The downregulation of FTO promotes the occurrence of ulcerative colitis (UC) by reducing the expression of CERS6 and exacerbates UC through m^6^A-dependent mechanisms (Ma et al., 2024).

METTL3‐mediated m^6^A methylation plays a direct and indispensable role in the inflammatory response (Hu and Lin, 2020). It has been reported that genetic deletion of METTL3 alleviates periodontal destruction by restraining the expression of Caspase‐1 and IL‐1β. Furthermore, coptisine chloride, a natural small molecule, was discovered to be a novel METTL3 inhibitor and has a therapeutic effect on periodontitis (Zhou et al., 2024). The increased expression of METTL3 in macrophages is associated with the development of colitis, and depletion of METTL3 in macrophages can protect mice from colitis induced by dextran sulfate sodium(Yin et al., 2024). Huang et al. (2022a) reported that knockdown of METTL3 expression attenuated the inflammatory response and decreased TNF-α, IL-1β, and IL-6 expression in cornea-infected mice (Huang et al., 2022a). METTL3 silencing alleviated renal inflammation and programmed cell death in tubular epithelial cells in response to stimulation with TNF-α and lipopolysaccharide (LPS), whereas METTL3 overexpression had the opposite effects (Wang et al., 2022). METTL3 can influence defensin expression through its methylation activity and thus protect against bacterial infections (Zong et al., 2021). METTL3-mediated m^6^A modifications play a significant role in liver damage in hepatitis B virus–infected mice (Cheng et al., 2022). In addition, METTL3 expression is upregulated in pneumonia patient serum and LPS-treated cells, and LPS-induced inflammatory injury is attenuated by inhibiting METTL3 expression (Li et al., 2021). In LPS-treated MODE-K cells, METTL3 knockdown promoted cell viability, inhibited cell apoptosis, decreased apoptotic caspase 3/9 cleavage, and decreased the levels of proinflammatory cytokines (IL-1β, TNF-α, IL-6, and IL-18) and inflammatory enzymes (Yang et al., 2022). A study in macrophages suggested that METTL3 may attenuate LPS-induced proinflammatory pathways and the production of reactive oxygen species. Knockout of METTL3 significantly increased the expression of IL-6, TNF-α, and NOX induced by LPS and further revealed that Pyk2 may be a target gene of Mettl3 that affects the inflammatory response (Cai et al., 2023). The total content of m^6^A and the expression of METTL3 and METTL14 are increased in periodontal ligament cells stimulated with LPS, and knocking out METTL3 or METTL14 inhibits the LPS-induced expression of IL-6 (Huang et al., 2024). The content of m^6^A and the level of METTL3 in patients with diabetic nephropathy are significantly increased, and METTL3 silencing reduces the levels of ROS, TNF-α and IL-6 in patients with diabetic nephropathy and alleviates renal injury (Wang et al., 2024a). Taken together, these findings indicate that METTL3‐mediated m^6^A modification may be a key factor in the regulation of immune processes and inflammatory responses.

However, different experimental results have shown that METTL3 is expressed at lower levels in the necrotic intestine of patients and in the small intestine of cecal ligation and puncture (CLP) model mice. Furthermore, the loss of METTL3 in CLP model mice triggers significantly greater expression of TNF-α and IL-18 and downregulates the expression of ZO-1 and claudin-1 (Shi et al., 2025). In a study in which palmatine (PAL) improved experimental colitis in rats, PAL significantly increased the expression levels of METTL3, METTL14, and ZO-1 and repaired intestinal barrier dysfunction (Ji et al., 2024). A previous study revealed that the protein expression of the methylase METTL3 was increased in both retinal pigment epithelium (RPE) and ARPE19 cells after LPS stimulation, and further research revealed decreased proliferation and tight junction protein expression and increased inflammatory factor secretion in METTL3-silenced RPE cells (Meng et al., 2022). These results may have occurred because m^6^A modification is regulated by a combination of writers, erasers, and readers (Zhang et al., 2023).

METTL3 has great potential as a therapeutic target for intestinal inflammation, and the small-molecule inhibitors STM2457 and Cpd-564 can both bind to METTL3 to exert inhibitory effects (Wang et al., 2022; Yankova et al., 2021). However, the advantages of traditional Chinese medicine, including the ease of material acquisition and application, suggest that developing monomeric compounds that target METTL3 may have substantial potential. Therefore, our goal was to identify the targeted inhibitory effects of traditional Chinese medicine monomers on METTL3. BBR and its analogs are METTL3 inhibitors that alleviate periodontitis by inhibiting pyroptosis (Zhou et al., 2024). In this study, BBR treatment alleviated the increase in m^6^A methylation levels and METTL3 protein expression caused by LPS challenge. Previous results also demonstrated that BBR exerts neuroprotective effects via the m^6^A methyltransferase METTL3, which regulates the NEAT1/miR-377-3p/Nampt axis in mouse astrocytes to ameliorate cerebral ischemia/reperfusion injury (Hu et al., 2024). In research on breast cancer, BBR treatment blocked breast cancer cell growth and metastasis partly by regulating METTL3-mediated m^6^A modification of FGF7 mRNA (Fu et al., 2024). Studies in acute kidney injury (AKI) have verified the inhibitory effect of BBR hydrochloride on METTL3 expression and demonstrated that BBR ameliorated folic acid-induced AKI and H_2_O_2_-induced pyroptosis in TCMK-1 cells by inhibiting METTL3 (Li et al., 2025). The alkaloid palmatine, which is also an active ingredient in Huanglian, has a similar effect on improving DSS-induced experimental colitis by regulating m^6^A methylation (Ji et al., 2024). As a novel small-molecule inhibitor of METTL3, BBR provides a potential route for the treatment of intestinal inflammation.

## Conclusions

Overall, we revealed that supplementation with 18 mg/kg BBR in the broiler diet can overcome LPS-induced intestinal mucosal damage by increasing the expression of tight junction proteins and exerting anti-inflammatory and antiapoptotic effects. This may be related to the inhibition of METTL3 protein expression and the reduction in m^6^A methylation levels. Our research results are expected to identify new targets for preventing intestinal inflammatory damage in broiler chickens.

## Availability of data and materials

The sequencing datasets in this study are available from the corresponding author upon reasonable request.

Supplementary data 1: HE staining observation of pathological changes.

Supplementary data 2: The pre experiment for concentration screening of LPS and BBR.

## CRediT authorship contribution statement

**Lin Yuan:** Writing – original draft, Project administration, Conceptualization. **Wanli Li:** Writing – review & editing, Validation, Supervision, Methodology. **Guoxi Li:** Writing – review & editing, Validation, Supervision, Methodology. **Wei Jin:** Software, Formal analysis, Data curation. **Bingxun Wang:** Software, Formal analysis, Data curation. **Shengli Li:** Writing – original draft, Investigation. **Haoyu Wang:** Writing – original draft, Investigation.

## Disclosures

We declare that we have no financial and personal relationships with other people or organizations that can inappropriately influence our work, and there is no professional or other personal interest of any nature or kind in any product, service or company that could be construed as influencing the content of this paper.

## Appendix Supplementary materials

Image, application 1

Image, application 2

## Footnotes

Supplementary material associated with this article can be found, in the online version, at doi:10.1016/j.psj.2025.105677.
