# Supplementary material for: Effect of berberine on LPS-induced intestinal epithelial injury and m6A methylation in broilers
Source: Poult Sci. 2025 Aug 12;104(11):105677. doi: 10.1016/j.psj.2025.105677 (PMC12391687; doi:10.1016/j.psj.2025.105677)
Supplement: Supplementary file 1 [file mmc1.docx]

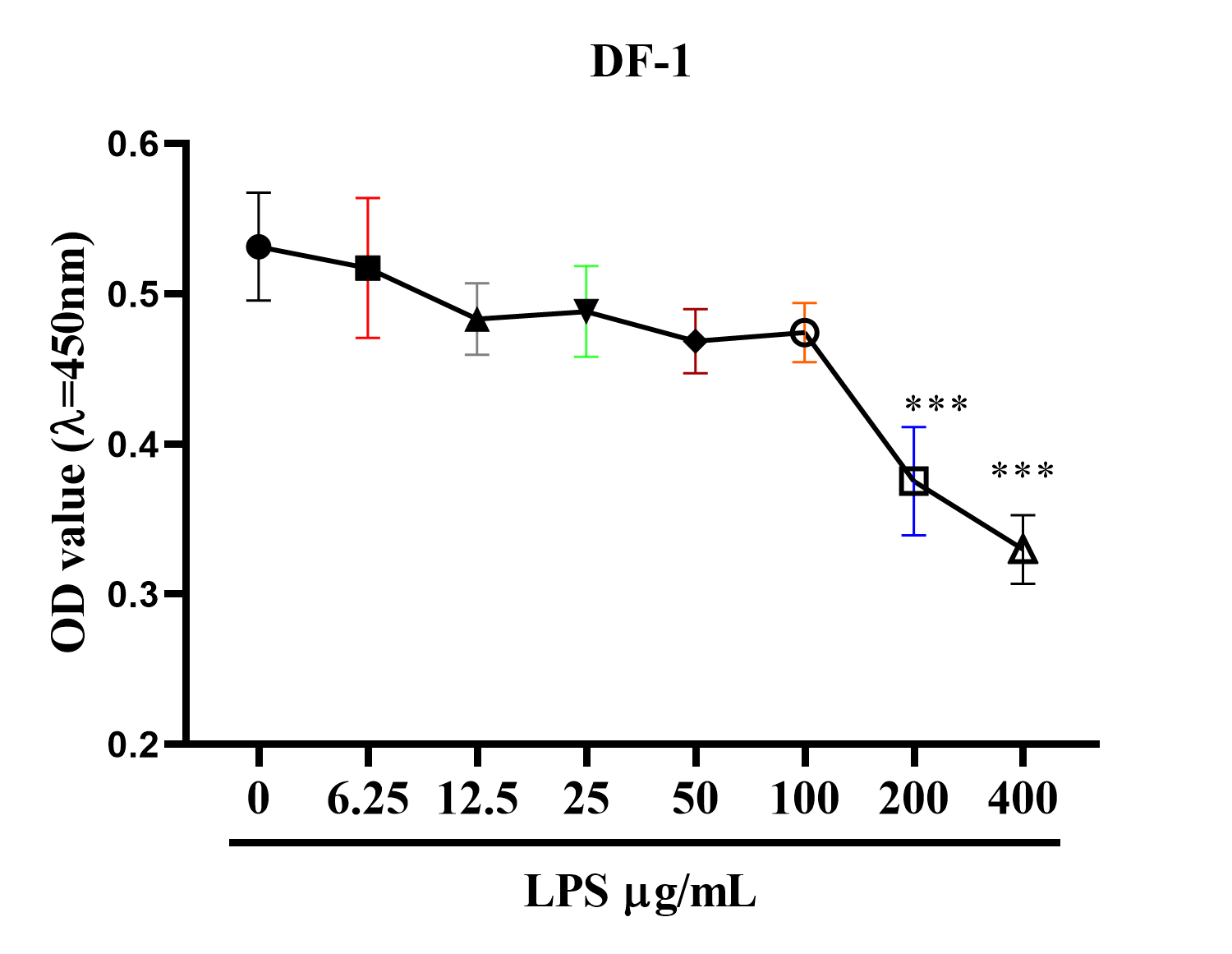


LPS had no significant effect on cell activity between 0-100 μ g/mL, but had a significant inhibitory effect on cell activity at 200 μ g/mL and 400 μg/mL.


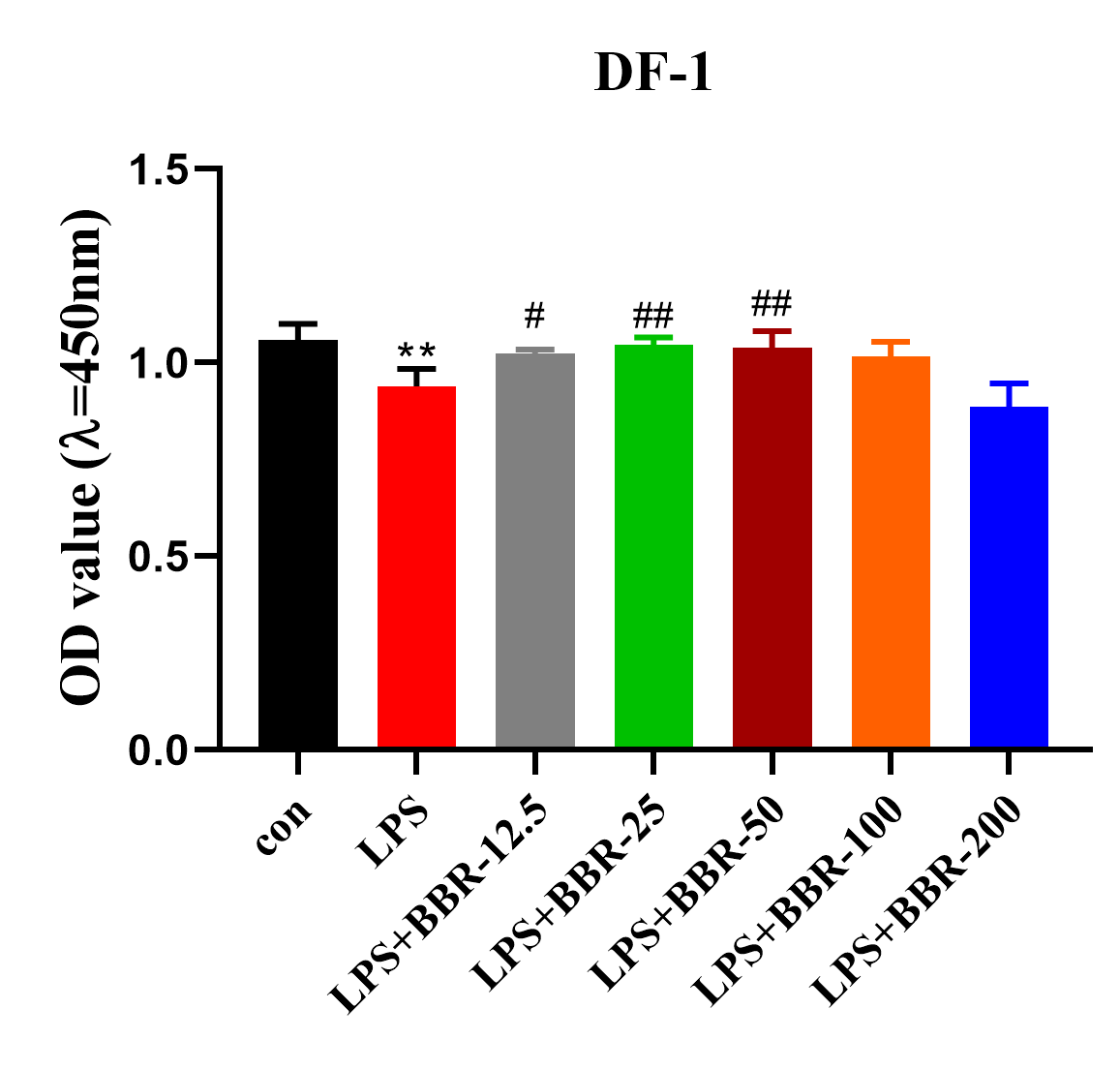


Compared with the con group, the activity of DF-1 cells in the LPS group was significantly reduced; Compared with the LPS group, the cell viability of the LPS+BBR-12.5, LPS+BBR-25, and LPS+BBR-50 groups was significantly increased, while there was no significant change in cell viability of the LPS+BBR-100 and LPS+BBR-200 groups.
