# Supplementary material for: Effect of berberine on LPS-induced intestinal epithelial injury and m6A methylation in broilers
Source: Poult Sci. 2025 Aug 12;104(11):105677. doi: 10.1016/j.psj.2025.105677 (PMC12391687; doi:10.1016/j.psj.2025.105677)
Supplement: Supplementary file 2 [file mmc2.docx]

HE staining observation of pathological changes

| 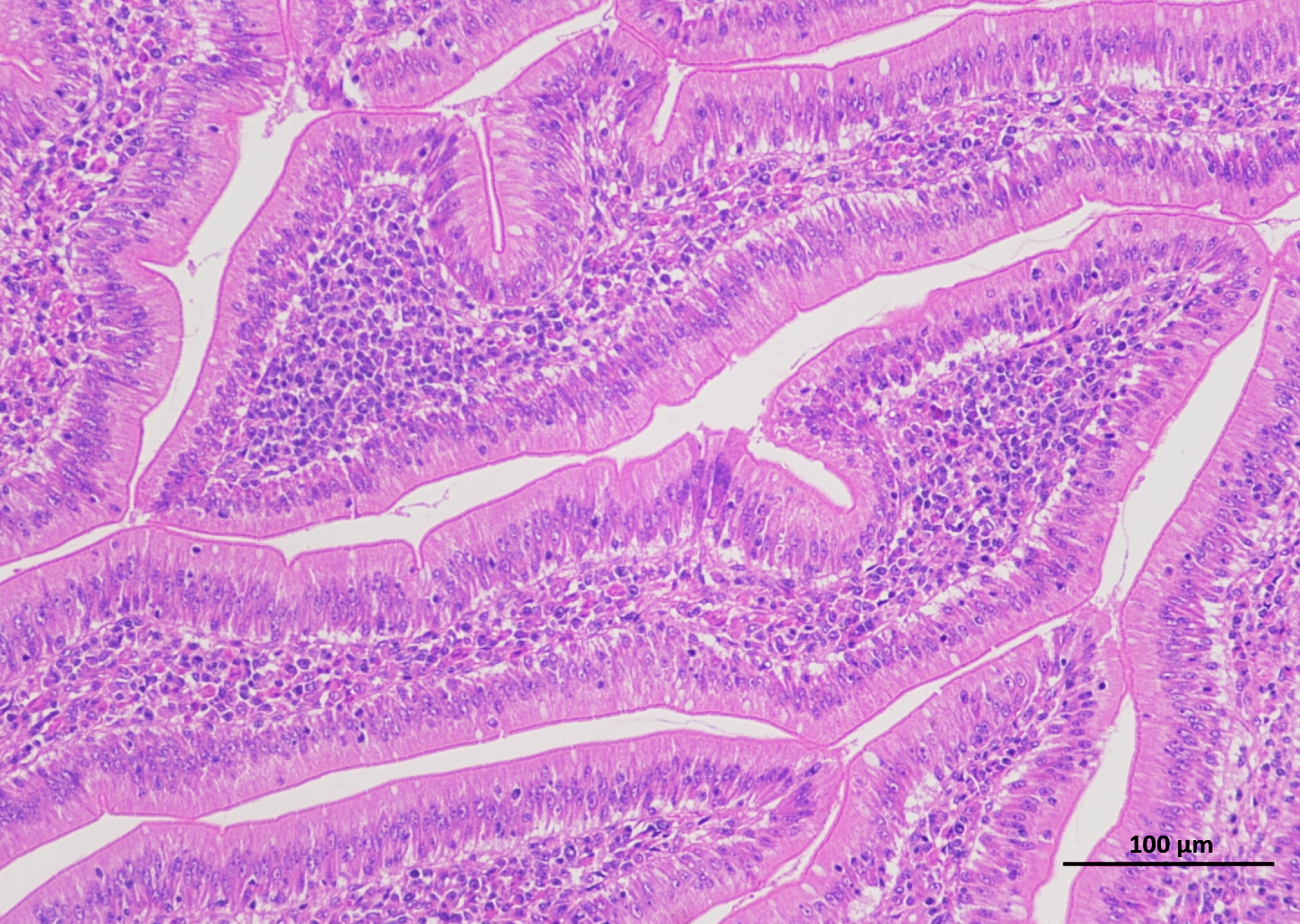 | 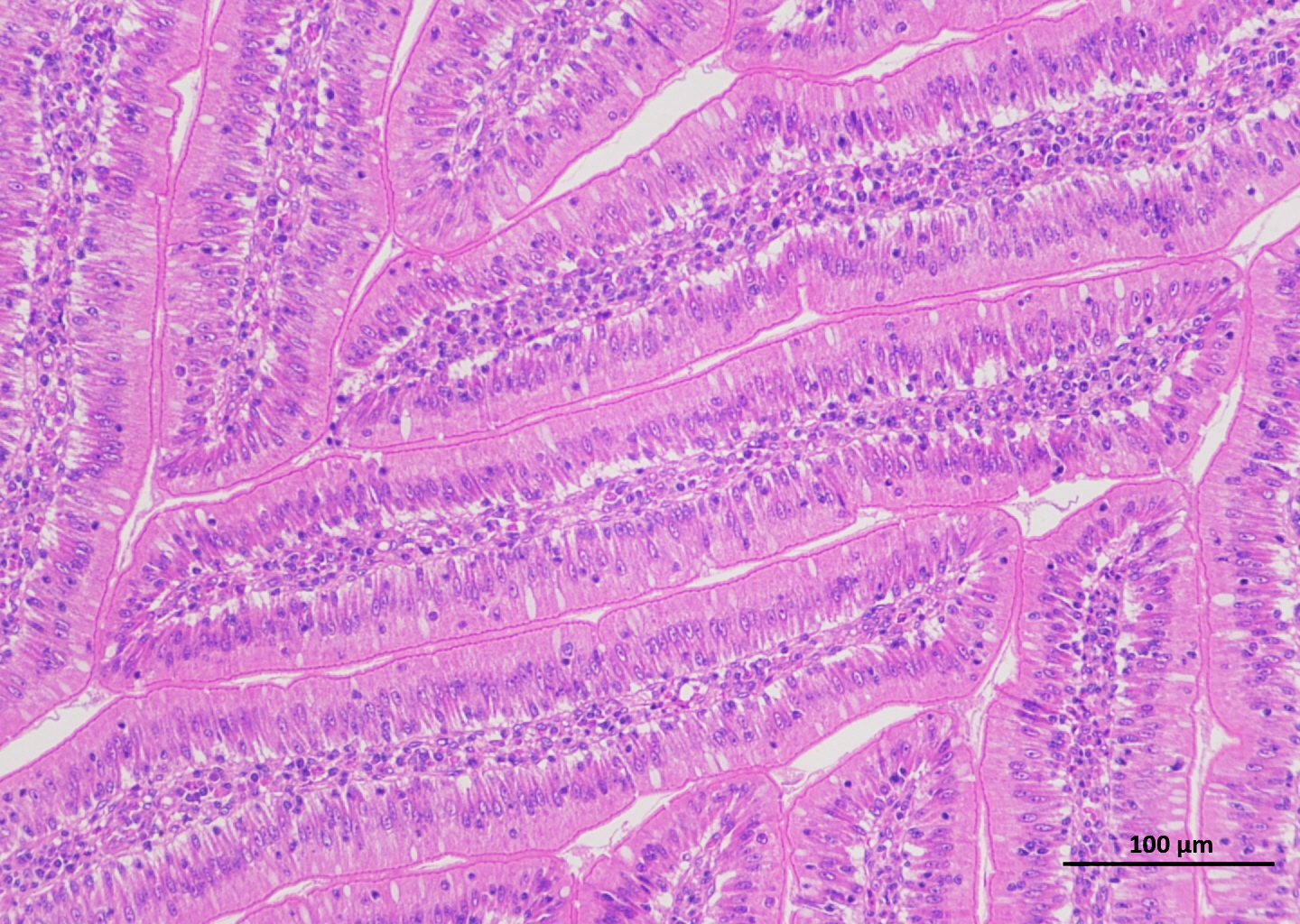 |
| --- | --- |
| NC-1 | NC-1 |
| The intestinal tissue shows a long length of villi, and the villi epithelium is mainly composed of a single layer of columnar epithelium and a small number of goblet cells; The lamina propria is composed of connective tissue and no other obvious abnormalities are observed. | |
| 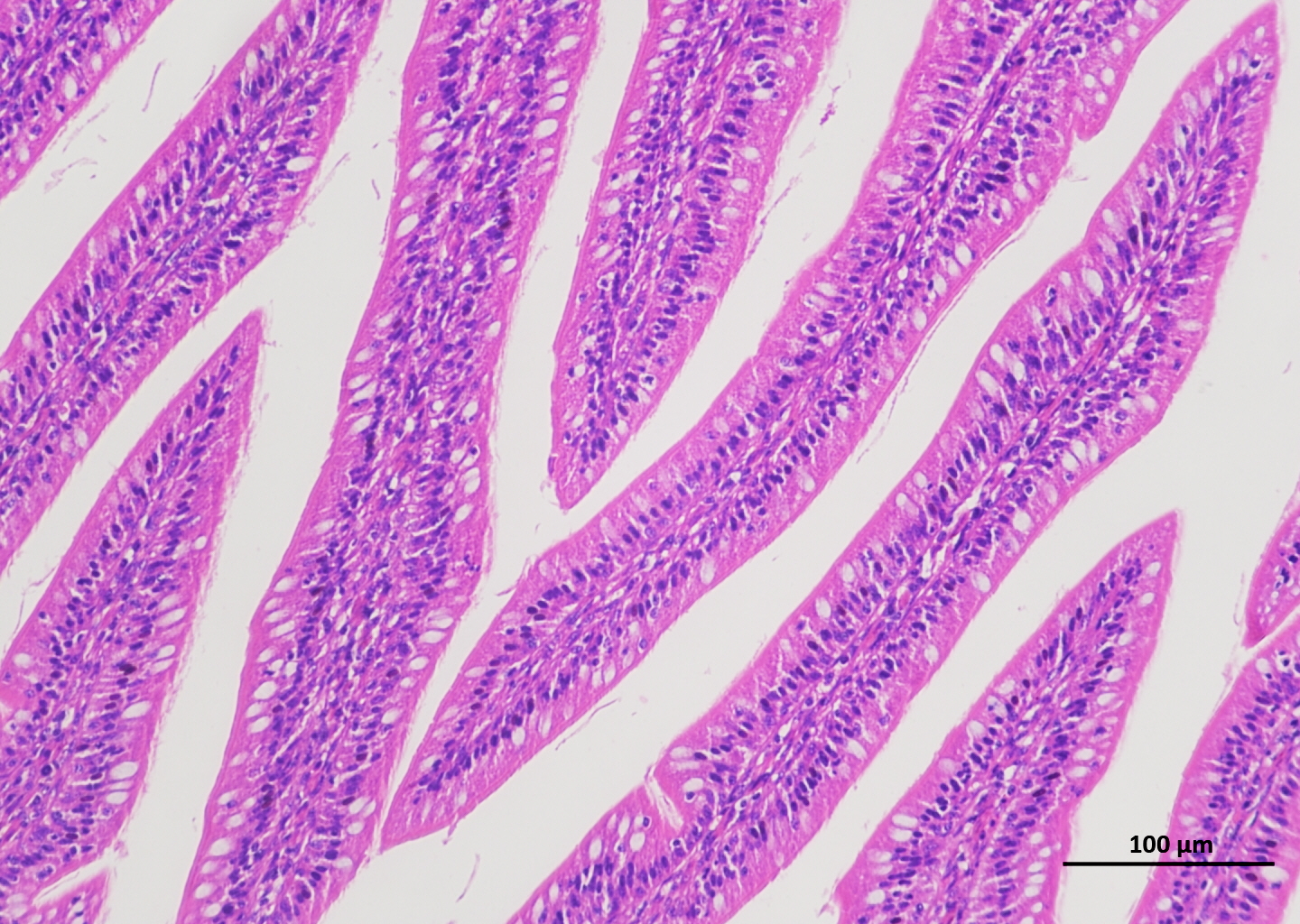 | 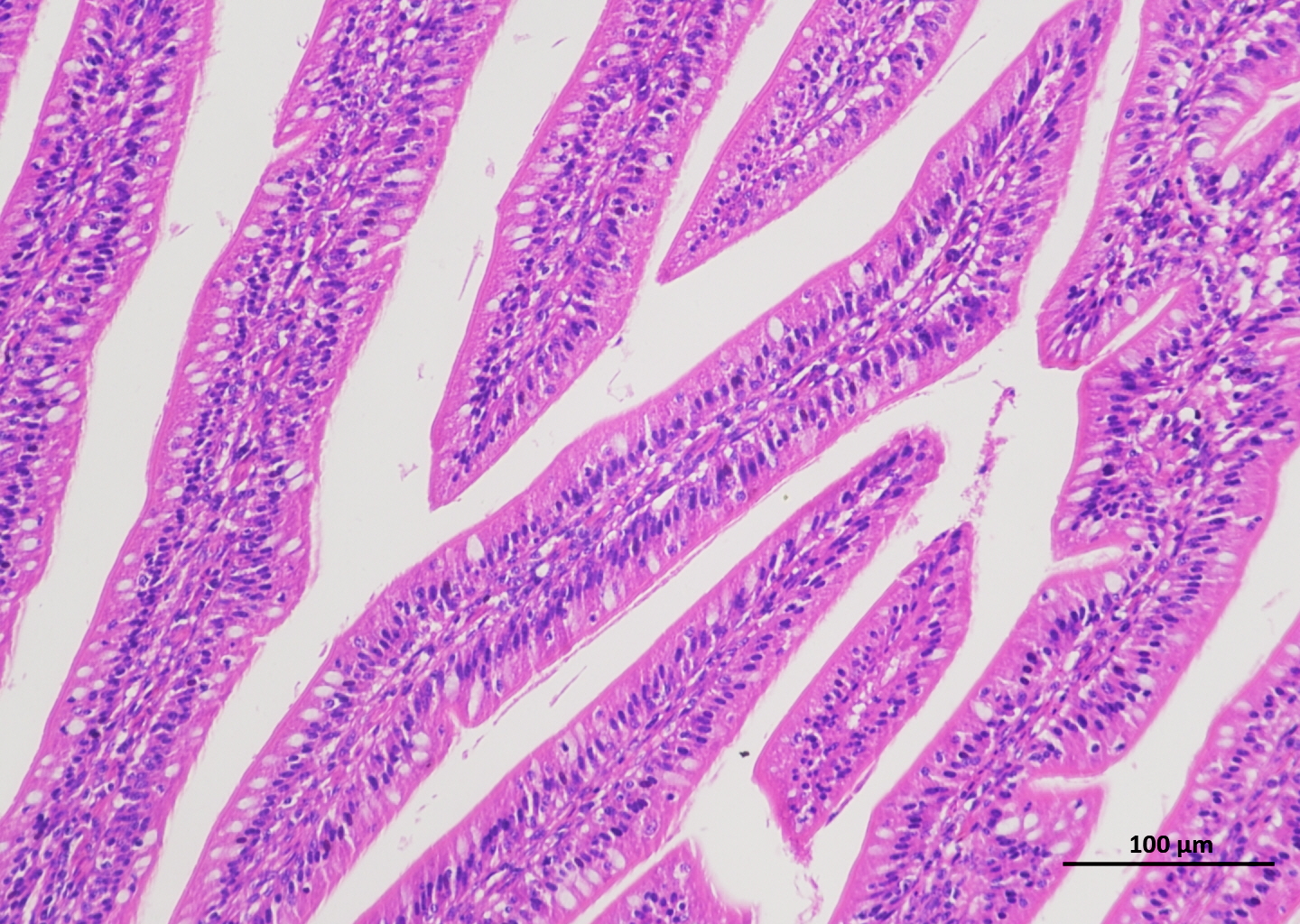 |
| NC-2 | NC-2 |
| The intestinal tissue shows a long length of villi, and the villi epithelium is mainly composed of a single layer of columnar epithelium and a large number of goblet cells; The lamina propria is composed of connective tissue and no other obvious abnormalities are observed. | |
| 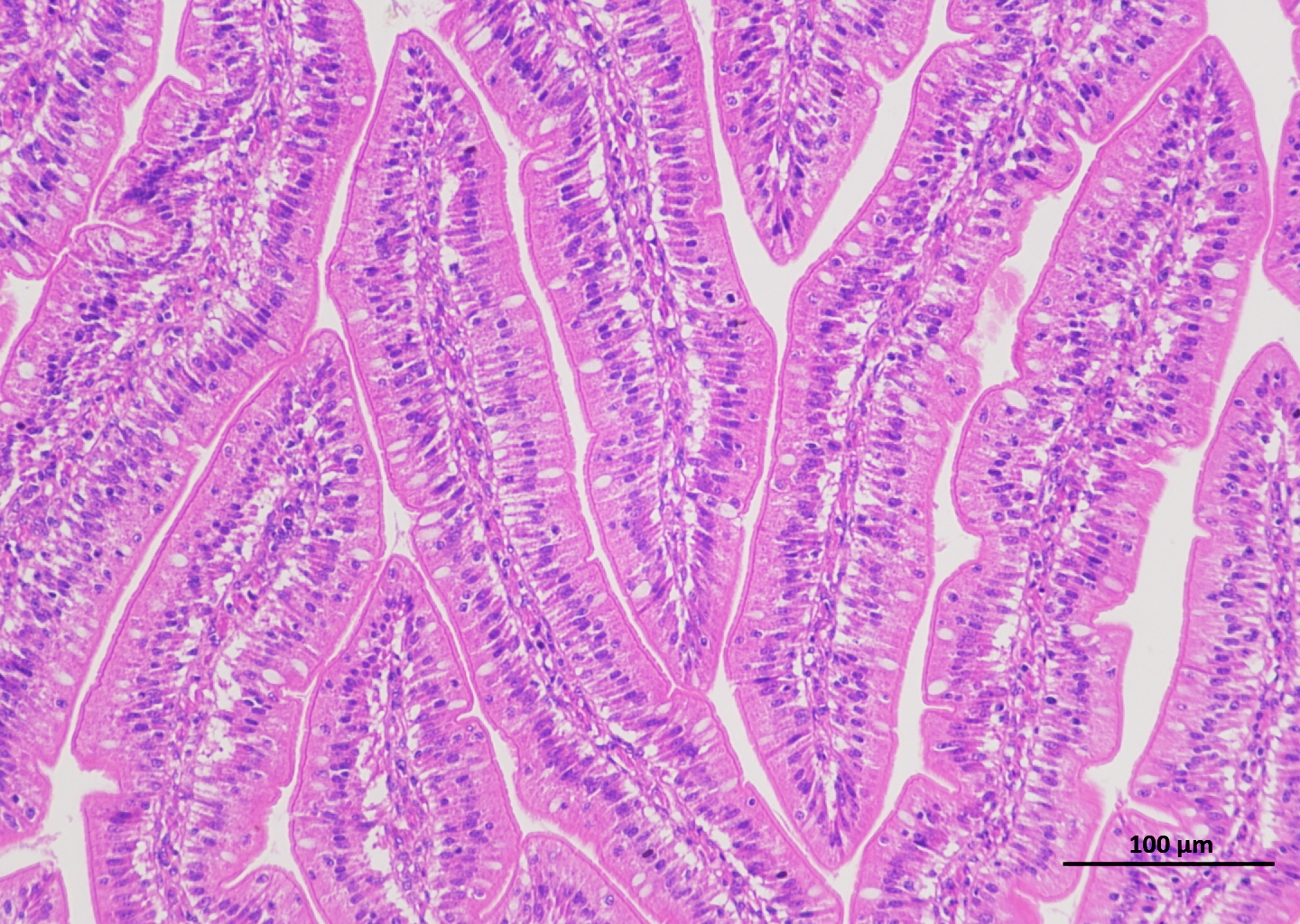 | 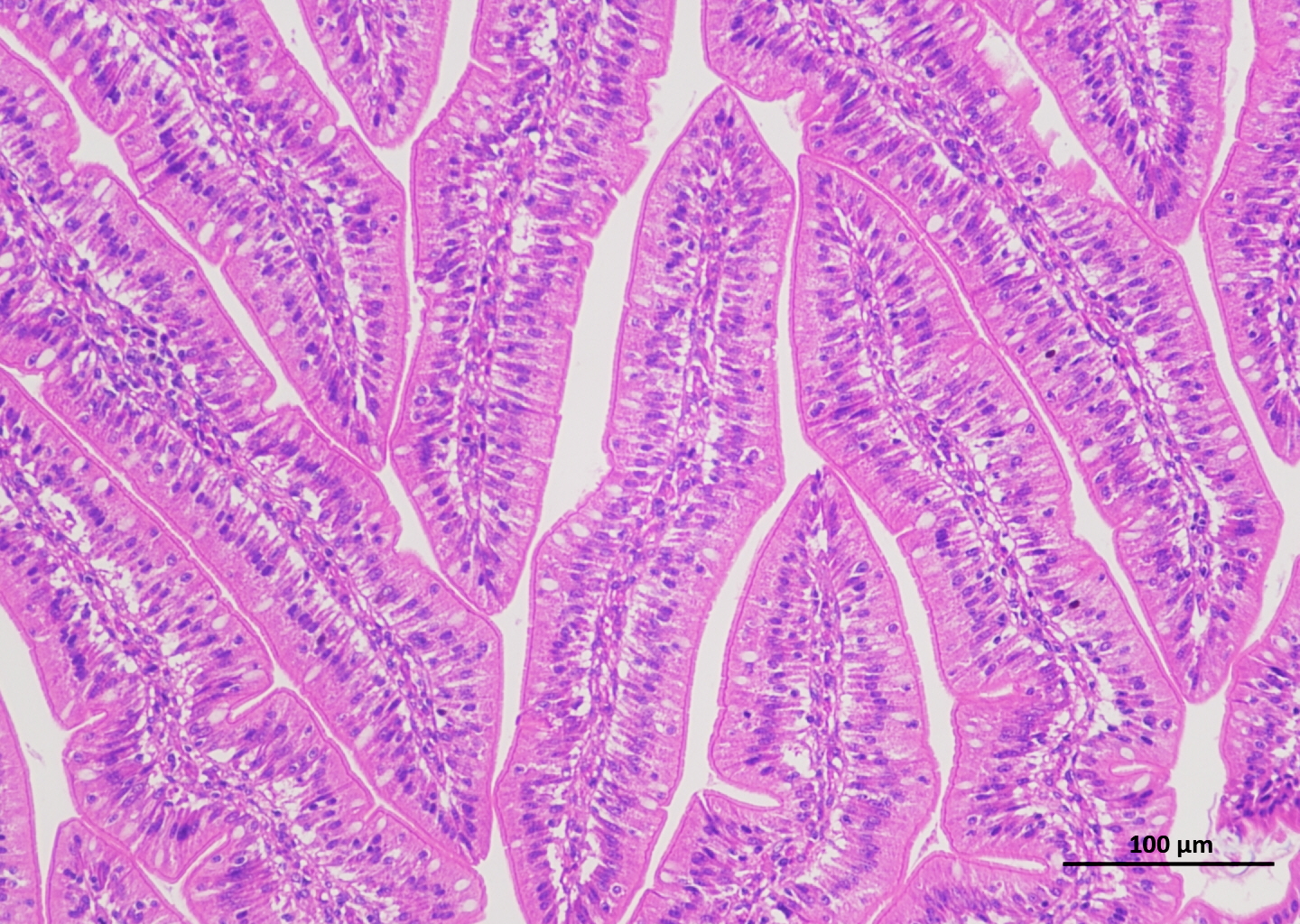 |
| NC-3 | NC-3 |
| The intestinal tissue shows a long length of villi, and the villi epithelium is mainly composed of a single layer of columnar epithelium and a large number of goblet cells; The lamina propria is composed of connective tissue and no other obvious abnormalities are observed. | |
| 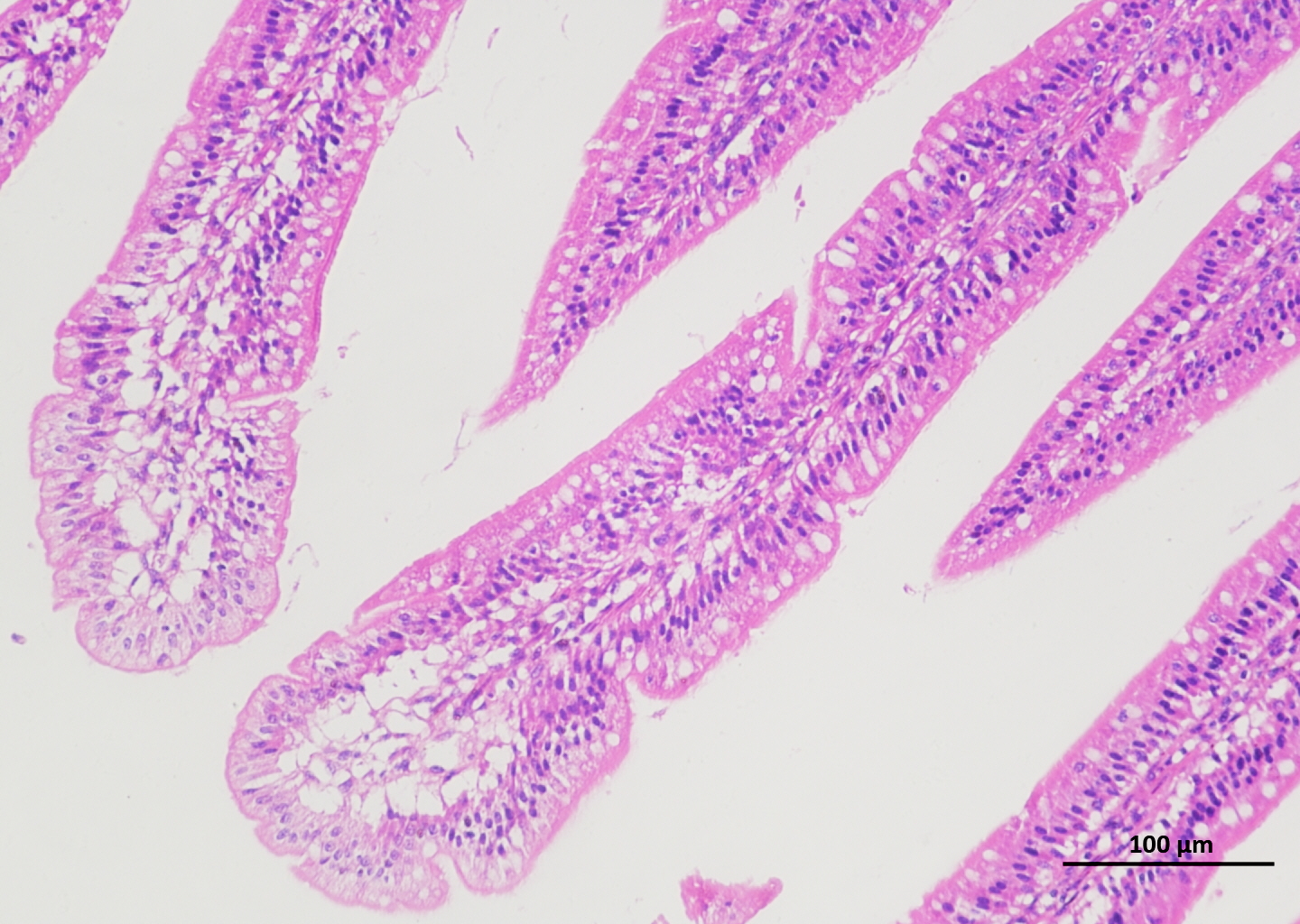 | 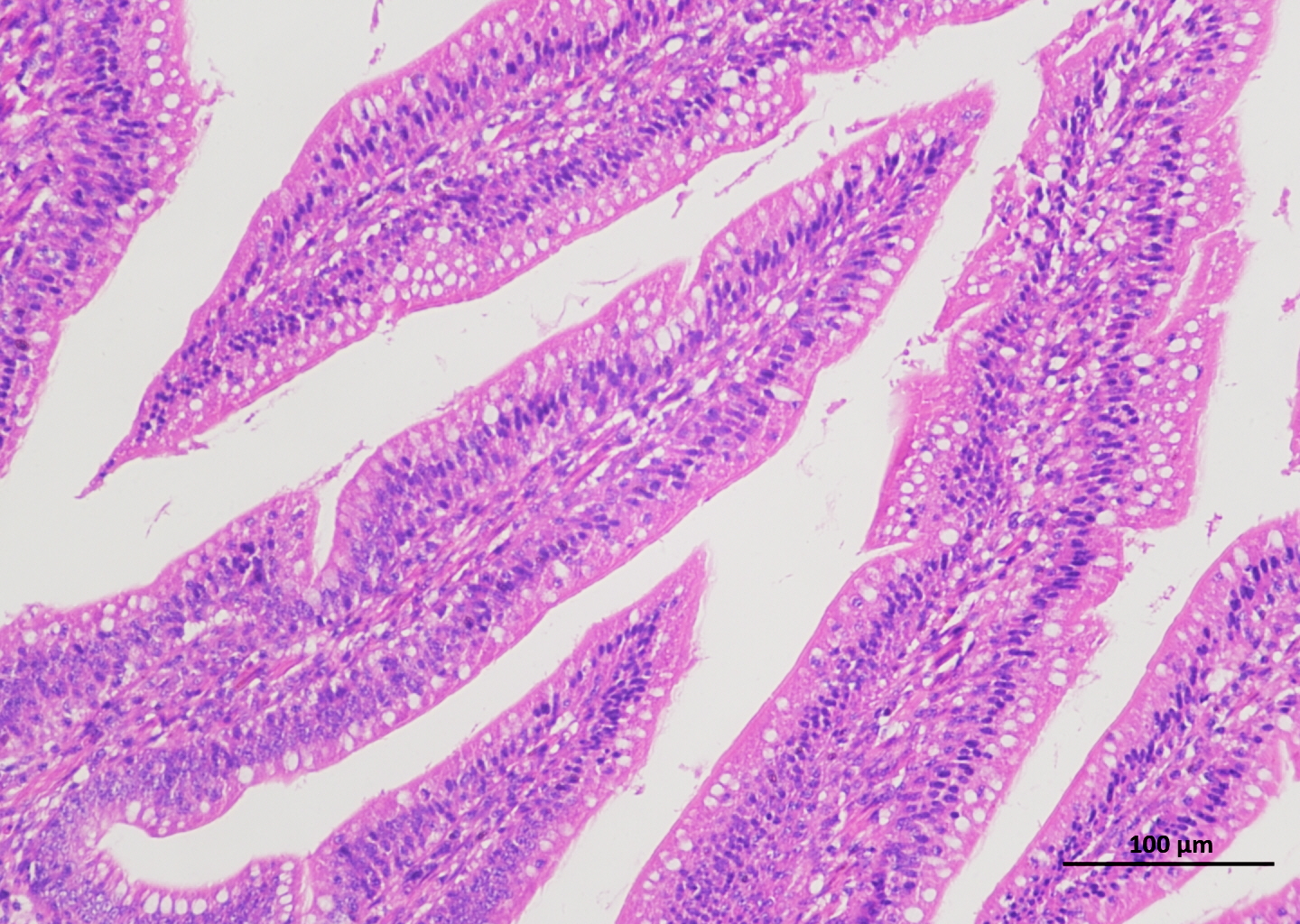 |
| T1-1 | T1-1 |
| The intestinal tissue shows a long length of villi, and the villi epithelium is mainly composed of a single layer of columnar epithelium and a large number of goblet cells; A small amount of intestinal villous epithelial cells show watery degeneration (red arrow), with swollen cells and loose and lightly stained cytoplasm; Occasionally, the top of the intestinal villous epithelium separates from the lamina propria, and the gap widens. A pale pink exudate (blue arrow) can be seen in the gap; The lamina propria is composed of connective tissue and no other obvious abnormalities are observed. | |
| 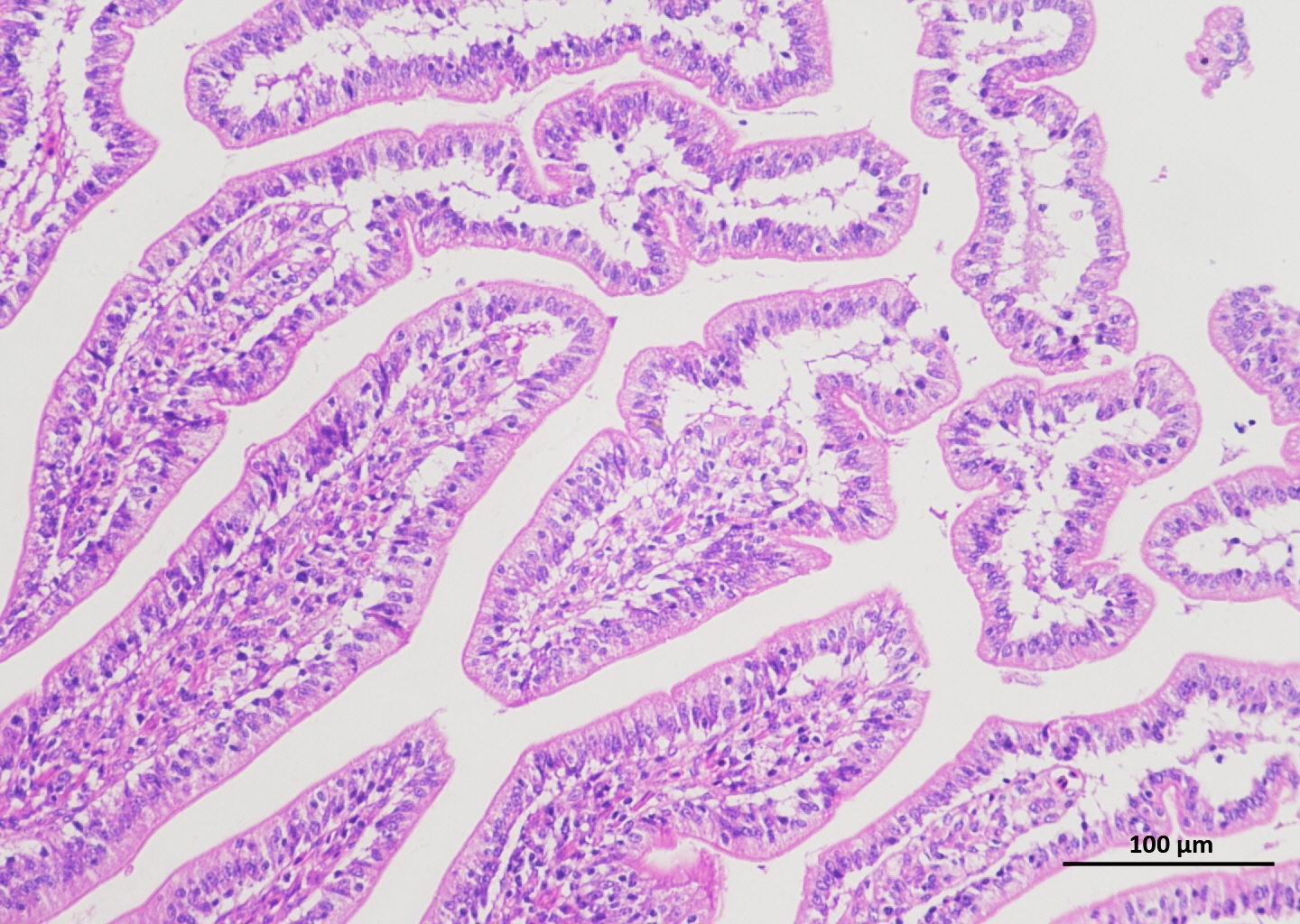 | 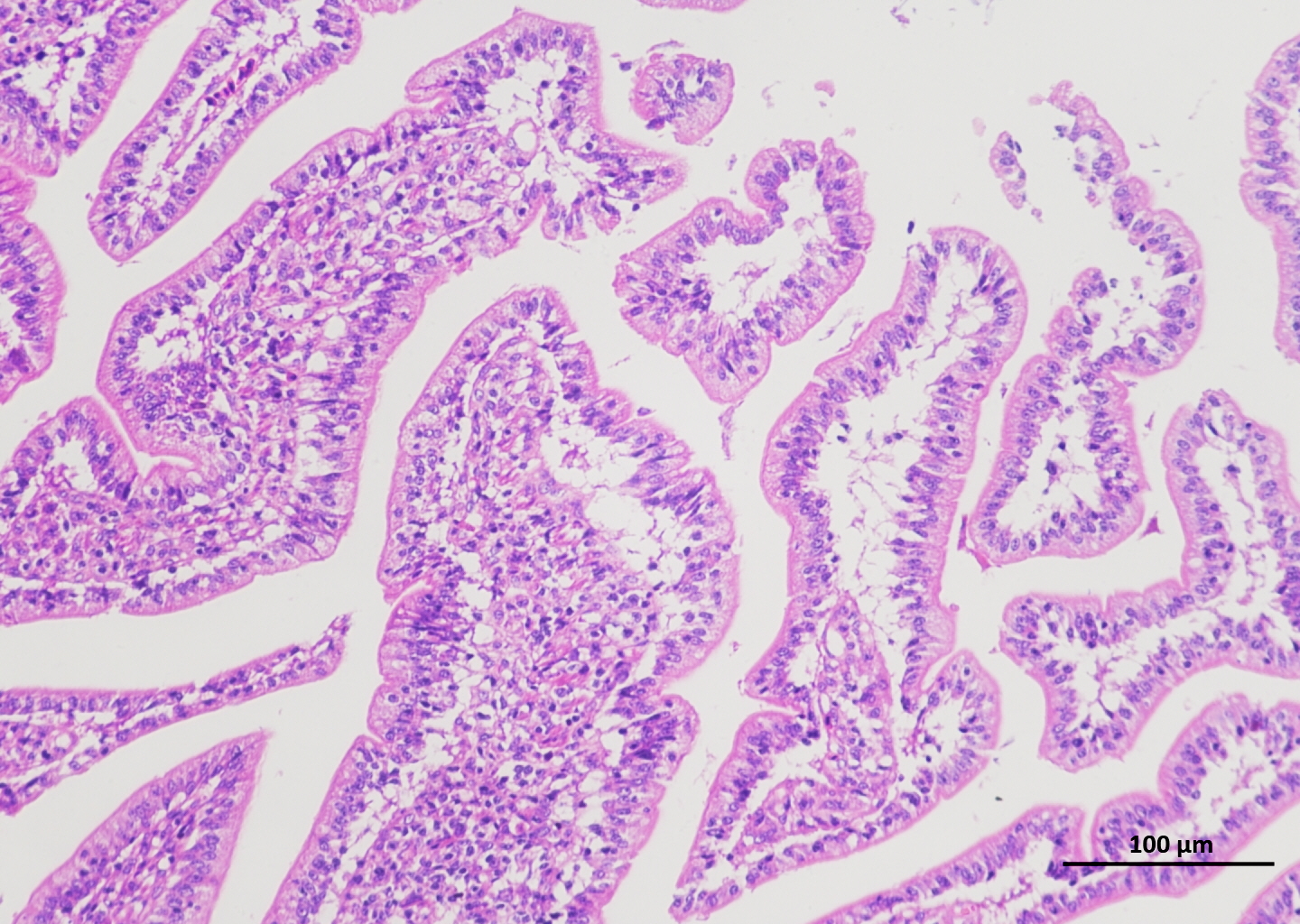 |
| T1-2 | T1-2 |
| Long and irregularly arranged villi can be seen in intestinal tissue, and the villi epithelium is mainly composed of a single layer of columnar epithelium and a small number of goblet cells; Multiple separation of intestinal villous epithelium and lamina propria (blue arrow), widening of gaps; A small amount of intestinal villous epithelial cells shed (yellow arrow); A small amount of intestinal villous epithelial cells show watery degeneration (red arrow), with swollen cells and loose and lightly stained cytoplasm; The lamina propria is composed of connective tissue and no other obvious abnormalities are observed. | |
| 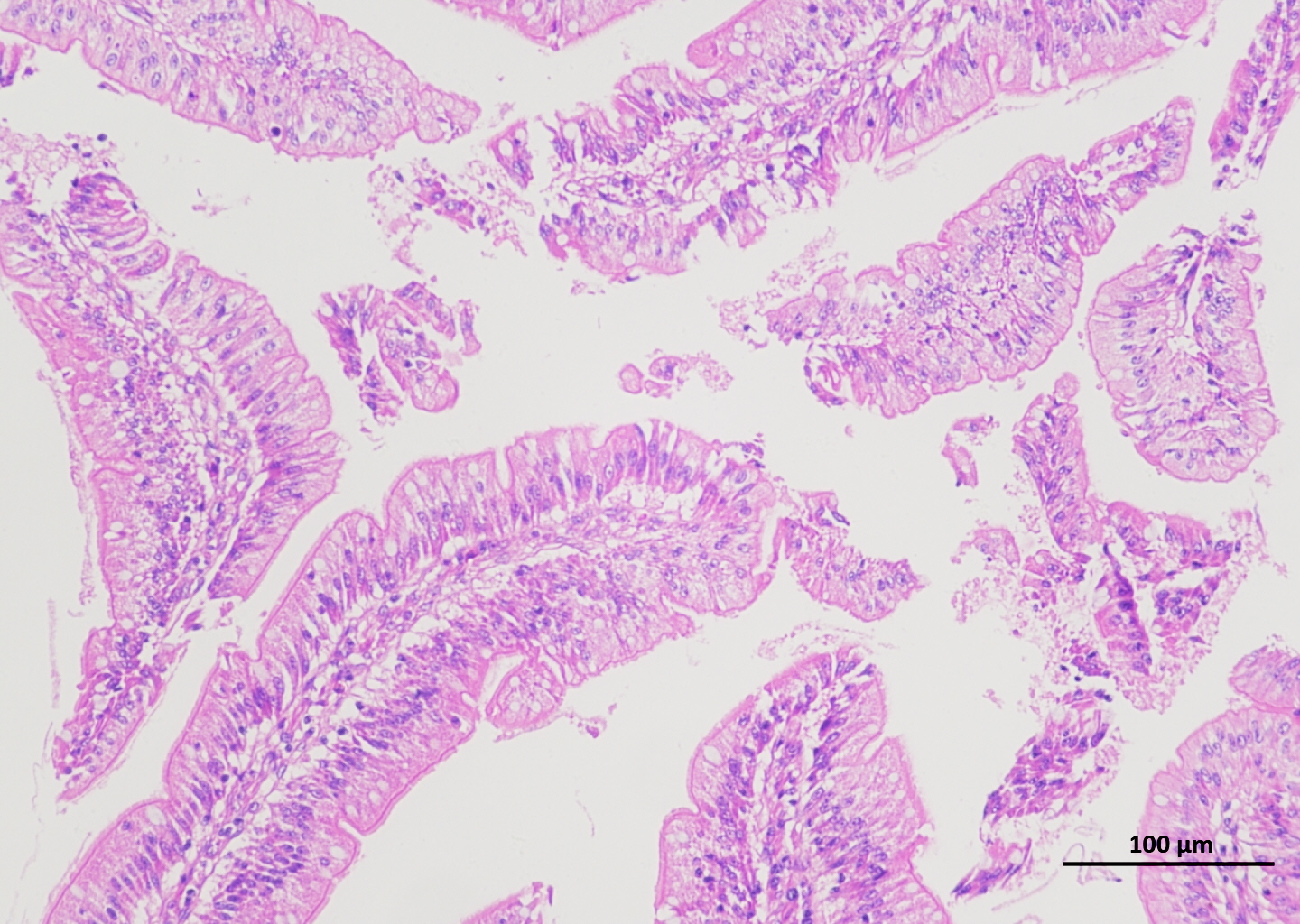 | 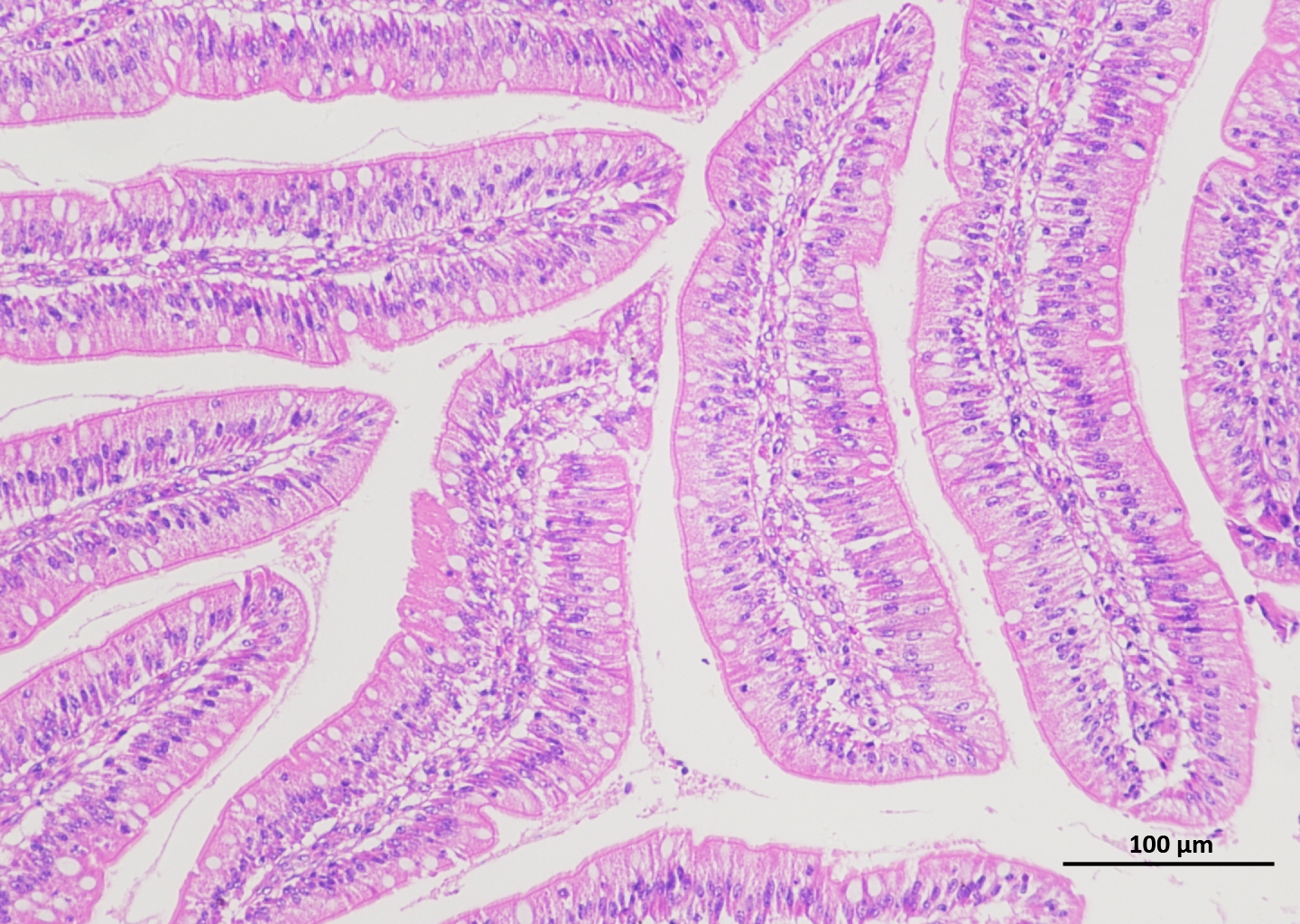 |
| T1-3 | T1-3 |
| The intestinal tissue shows a long length of villi, and the villi epithelium is mainly composed of a single layer of columnar epithelium and a large number of goblet cells; More intestinal villous epithelial cells have watery degeneration (red arrow), swollen cells, and loose and lightly stained cytoplasm; A small amount of intestinal villous epithelial cells shed (yellow arrow); The lamina propria is composed of connective tissue and no other obvious abnormalities are observed. | |
| 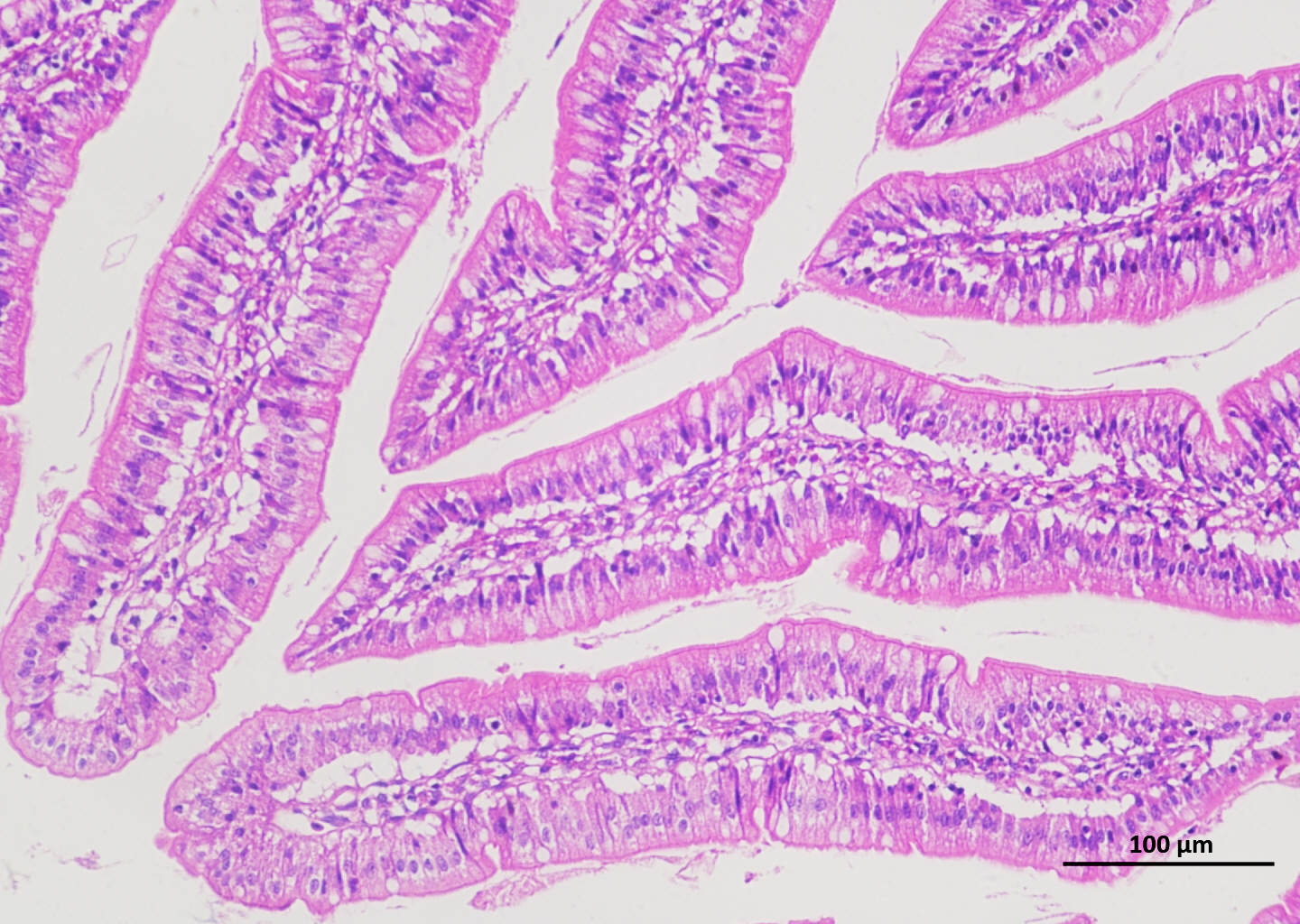 | 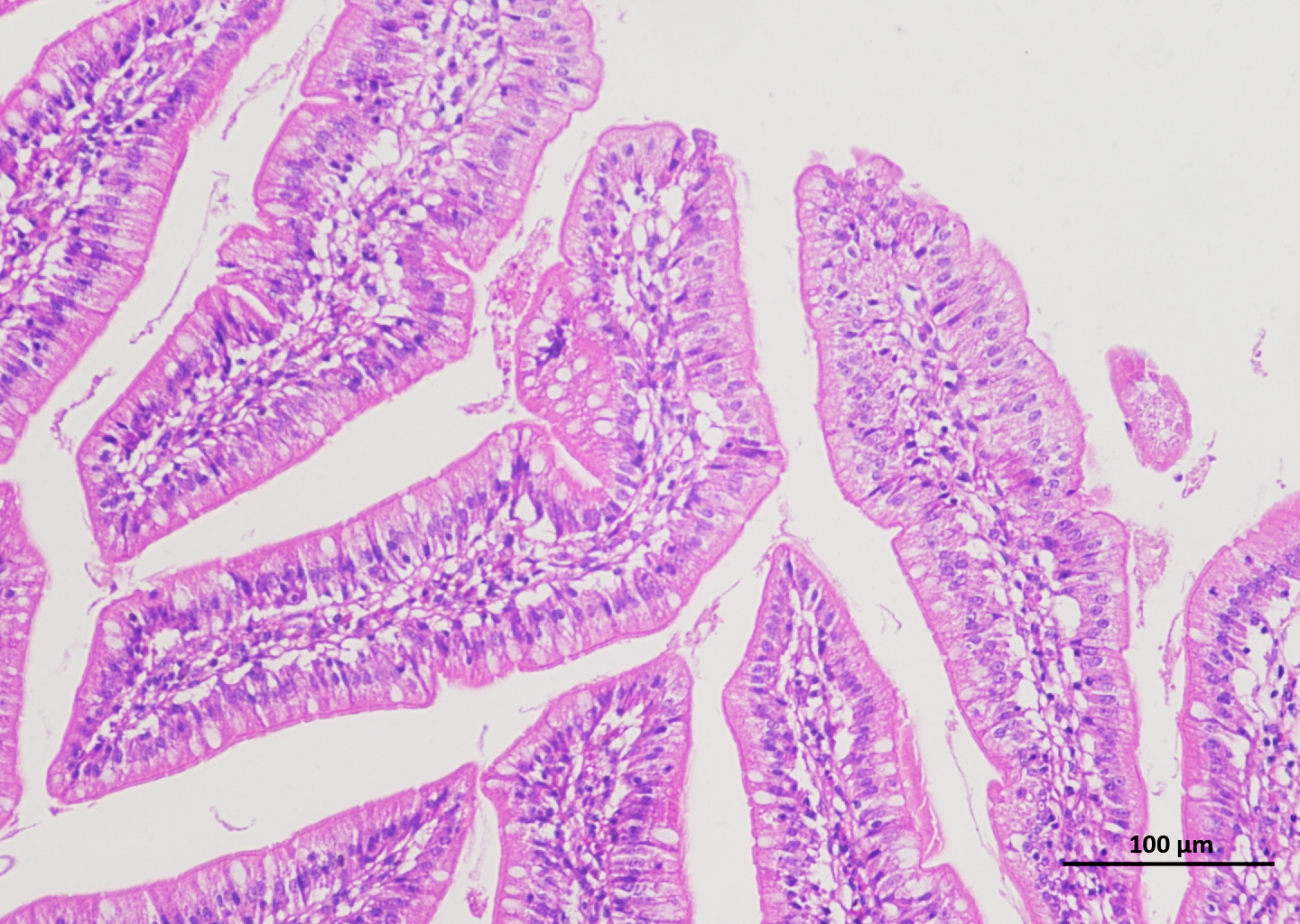 |
| T2-1 | T2-1 |
| The intestinal tissue shows a long length of villi, and the villi epithelium is mainly composed of a single layer of columnar epithelium and a large number of goblet cells; More intestinal villous epithelial cells have watery degeneration (red arrow), swollen cells, and loose and lightly stained cytoplasm; The lamina propria is composed of connective tissue and no other obvious abnormalities are observed. | |
| 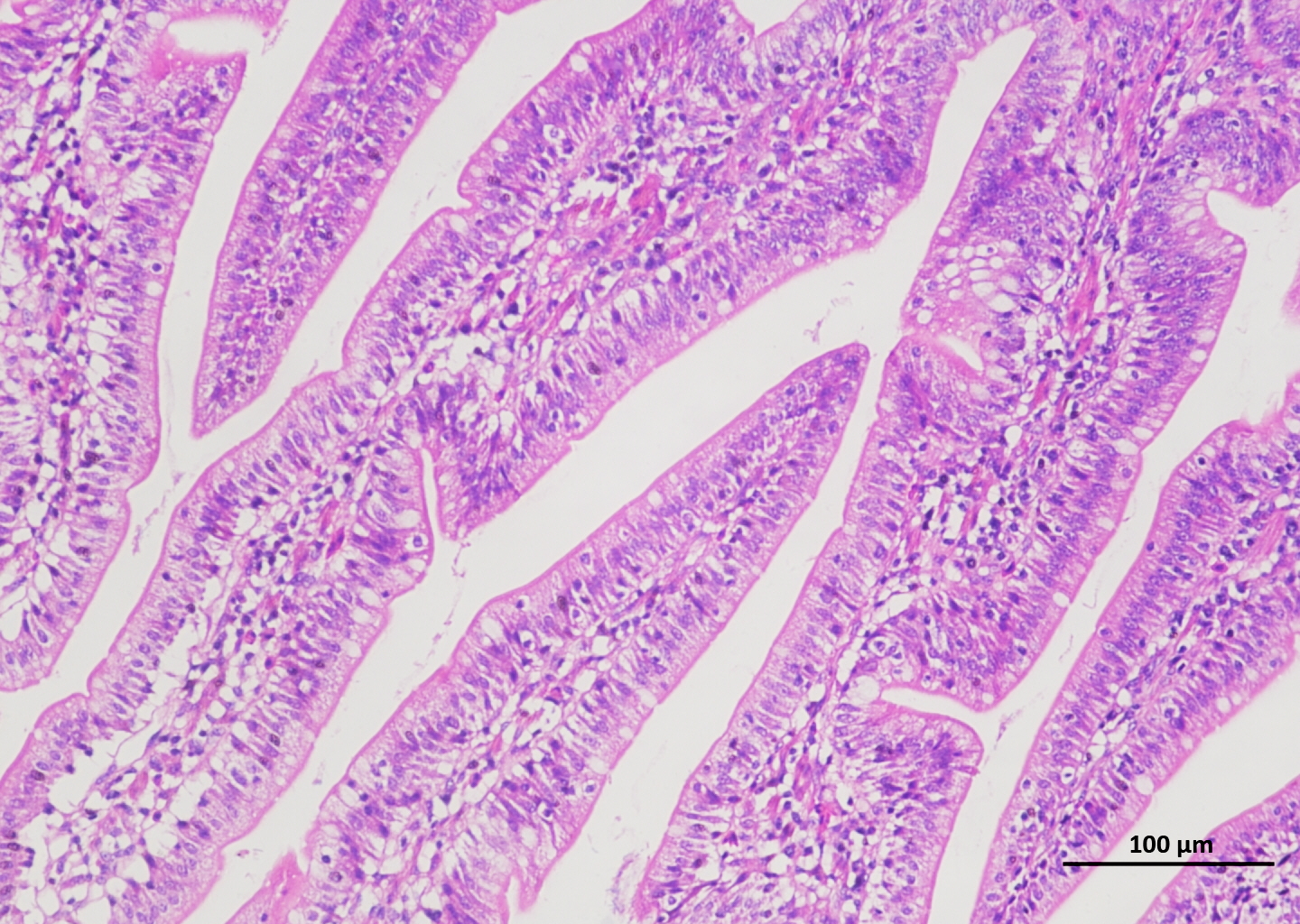 | 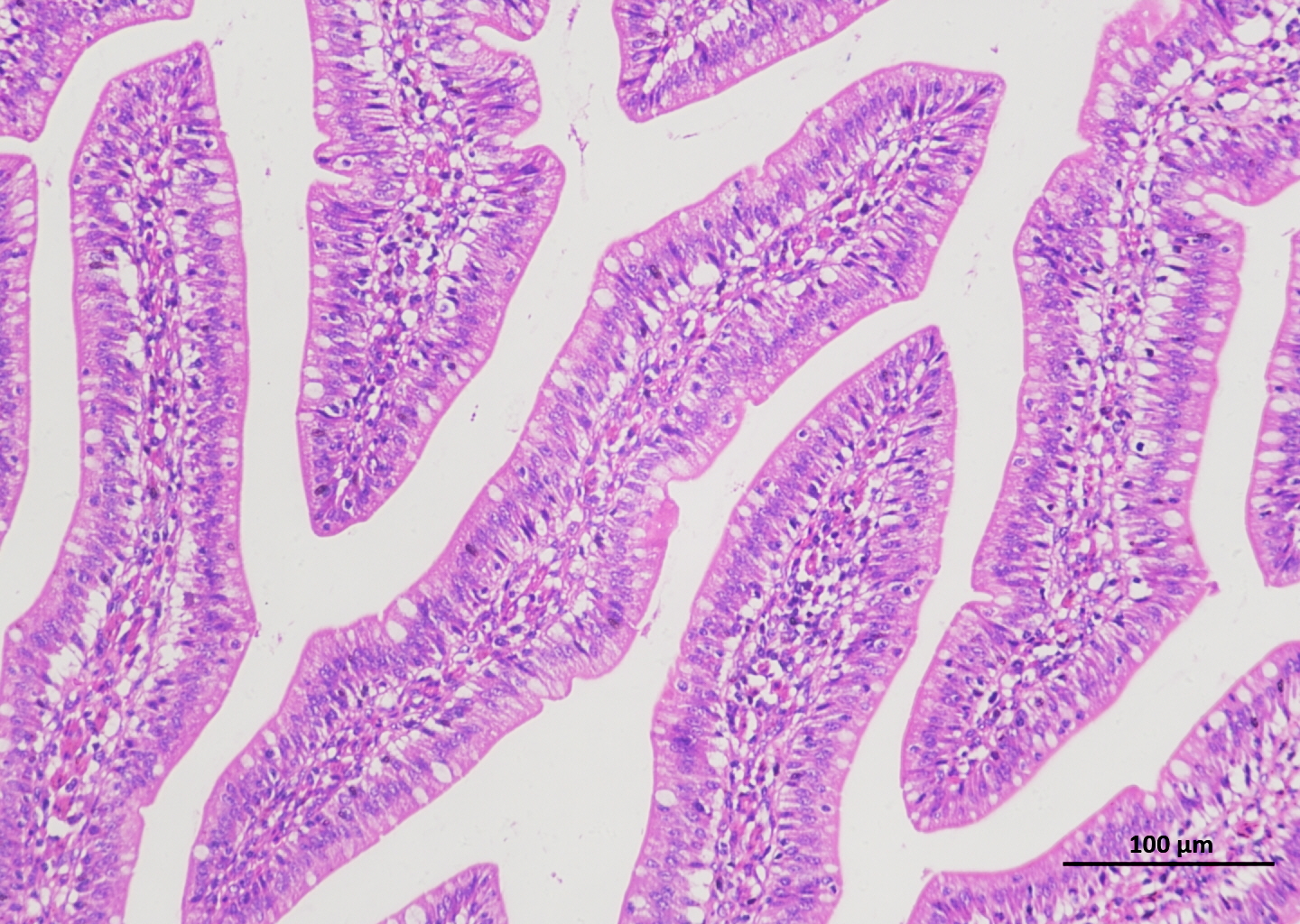 |
| T2-2 | T2-2 |
| The intestinal tissue shows a long length of villi, and the villi epithelium is mainly composed of a single layer of columnar epithelium and a large number of goblet cells; A small amount of intestinal villous epithelial cells show watery degeneration (red arrow), with swollen cells and loose and lightly stained cytoplasm; Occasional separation of intestinal villous epithelium and lamina propria (blue arrow) with widened gaps; The lamina propria is composed of connective tissue and no other obvious abnormalities are observed. | |
| 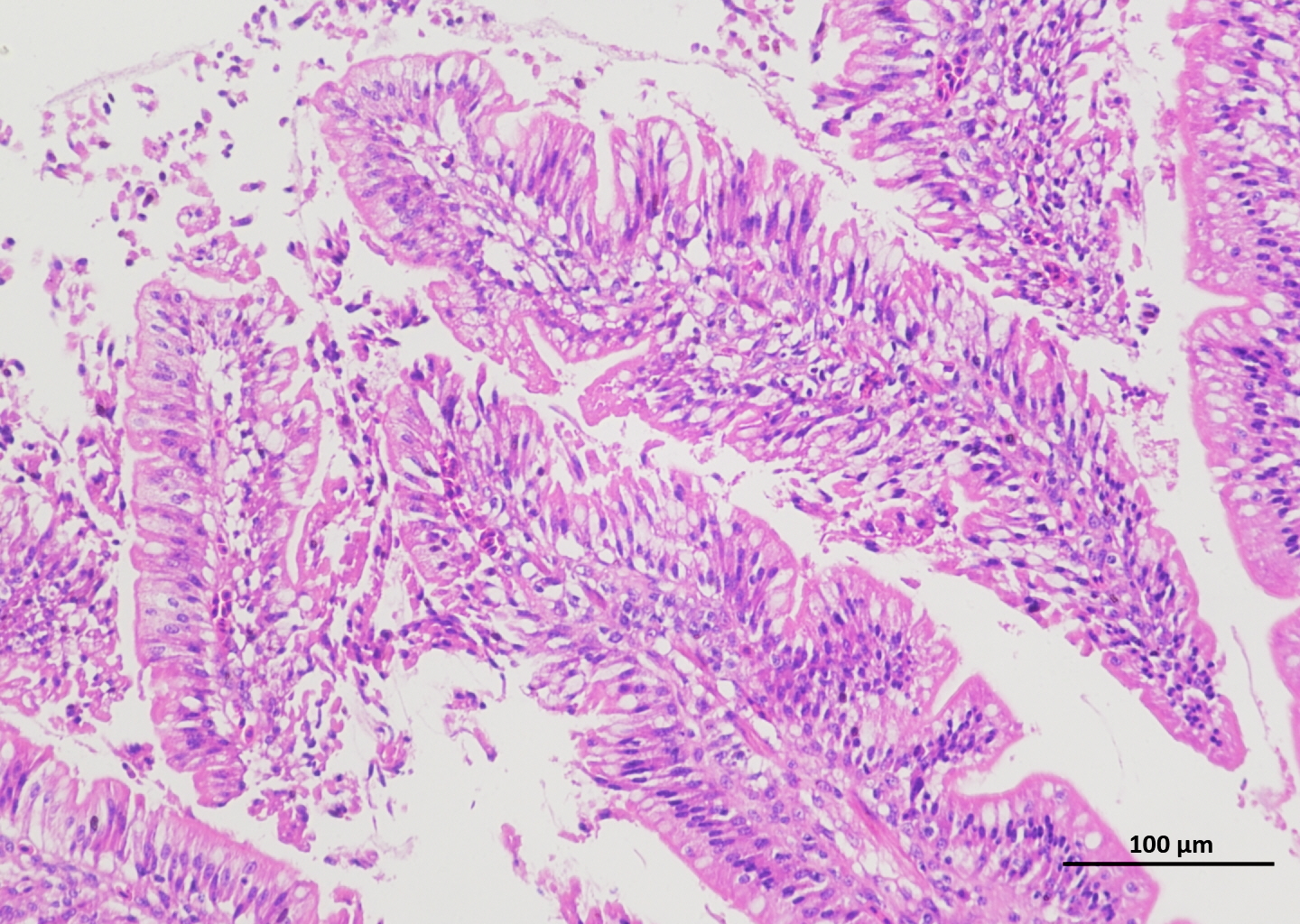 | 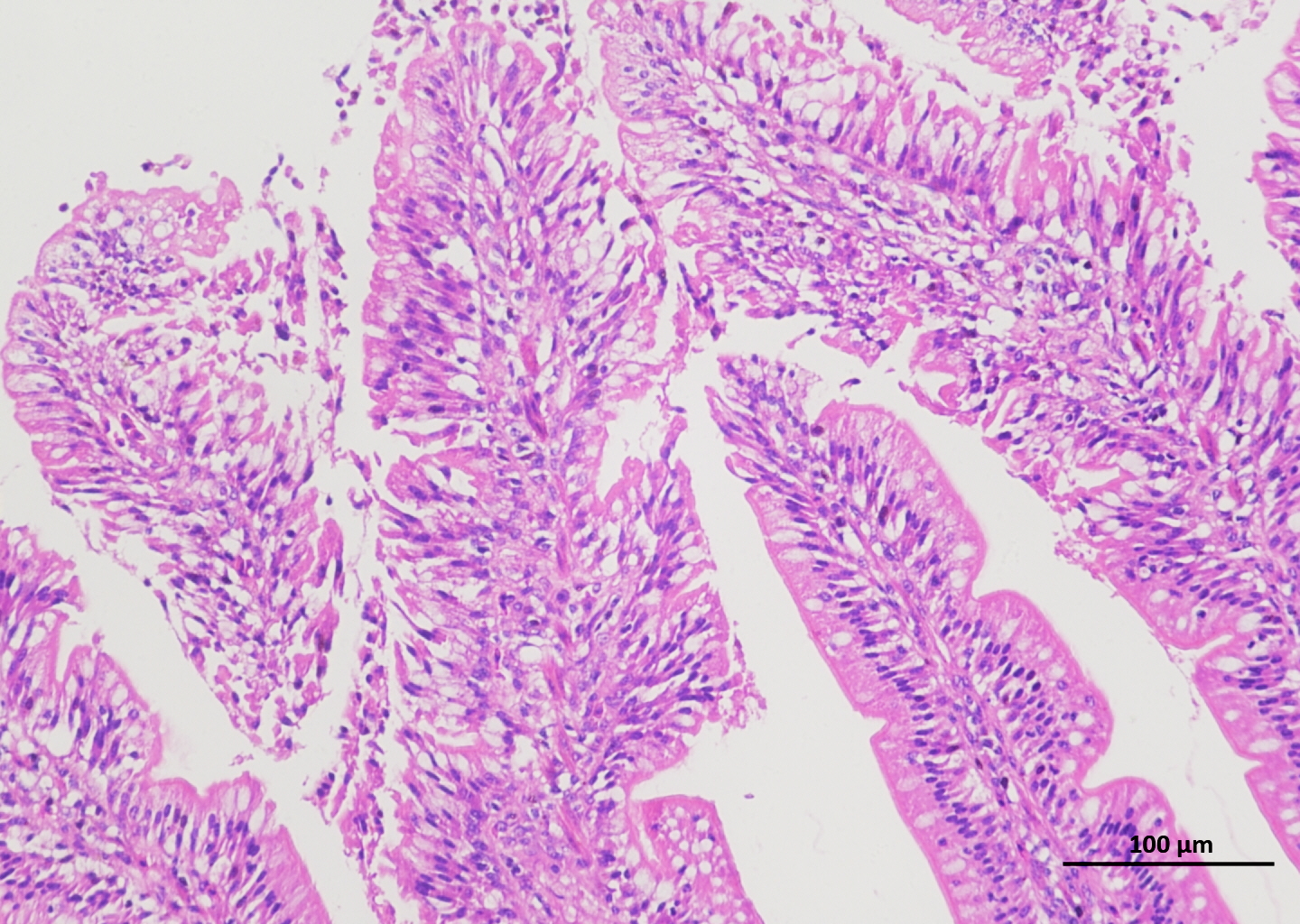 |
| T2-3 | T2-3 |
| The intestinal tissue shows a long length of villi, and the villi epithelium is mainly composed of a single layer of columnar epithelium and a large number of goblet cells; More intestinal villous epithelial cells shed (yellow arrow); A small amount of intestinal villous epithelial cells show watery degeneration (red arrow), with swollen cells and loose and lightly stained cytoplasm; The lamina propria is composed of connective tissue and no other obvious abnormalities are observed. | |
| 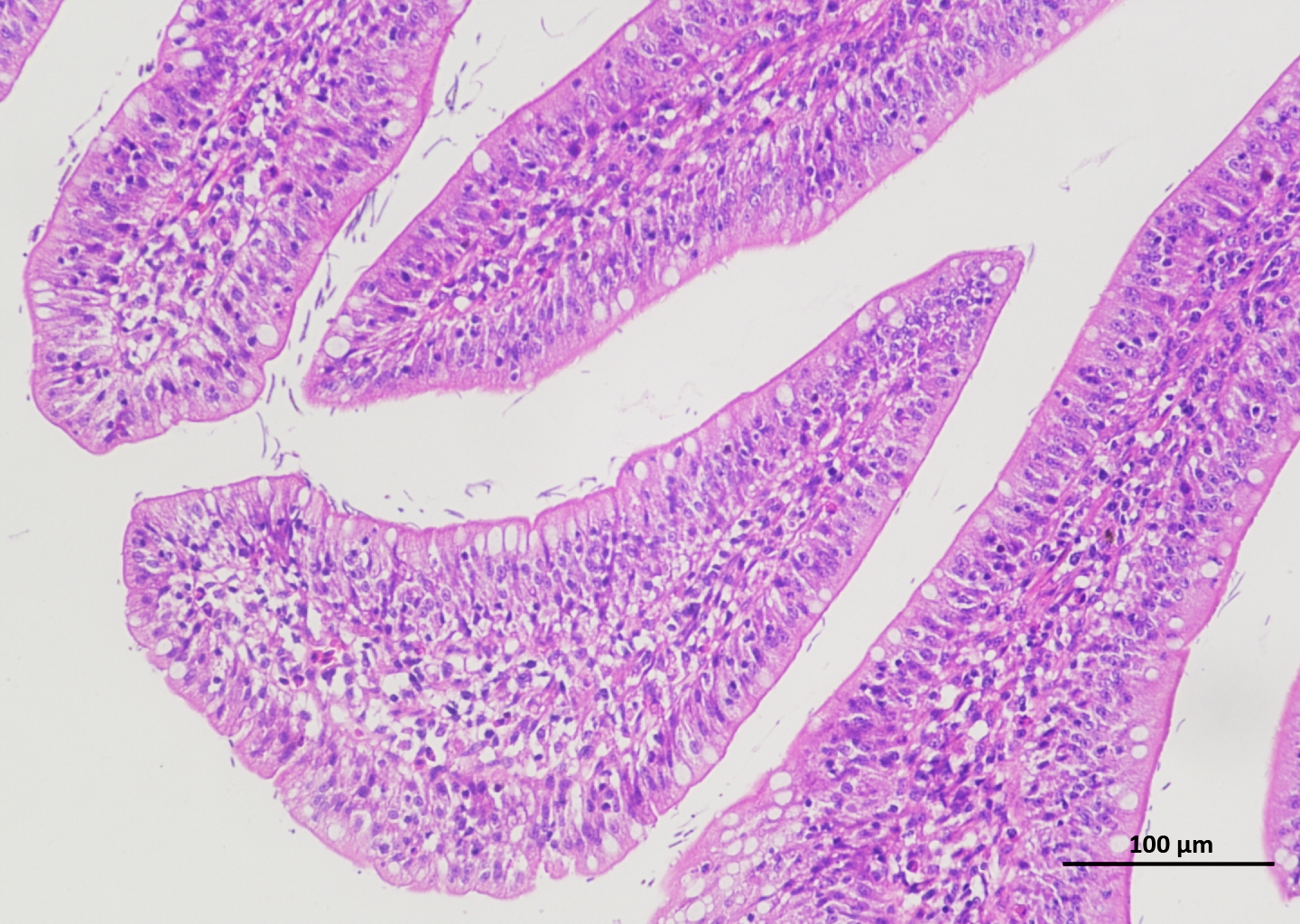 | 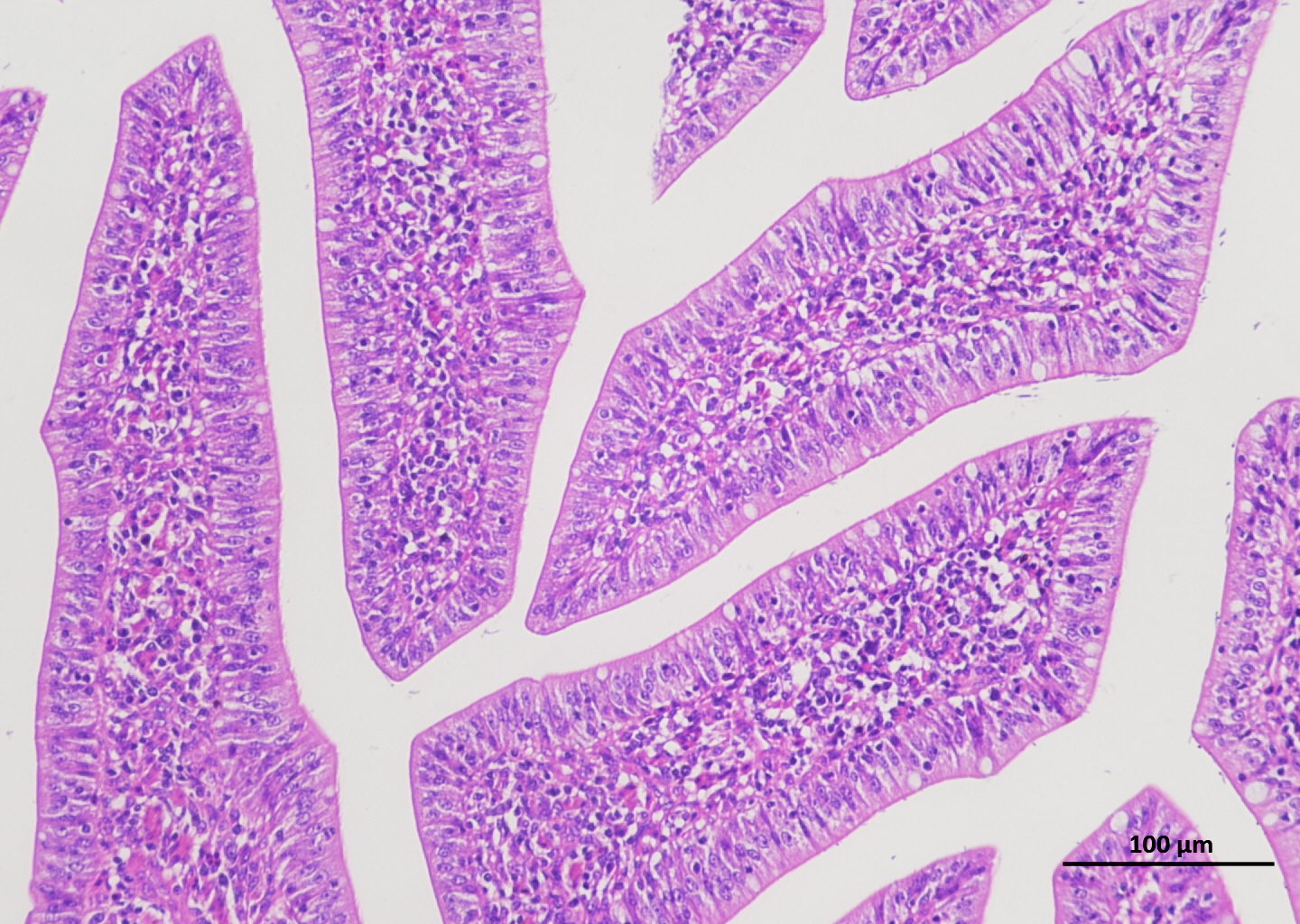 |
| T3-1 | T3-1 |
| The intestinal tissue shows a long length of villi, and the villi epithelium is mainly composed of a single layer of columnar epithelium and a small number of goblet cells; A small amount of intestinal villous epithelial cells show watery degeneration (red arrow), with swollen cells and loose and lightly stained cytoplasm; The lamina propria is composed of connective tissue and no other obvious abnormalities are observed. | |
| 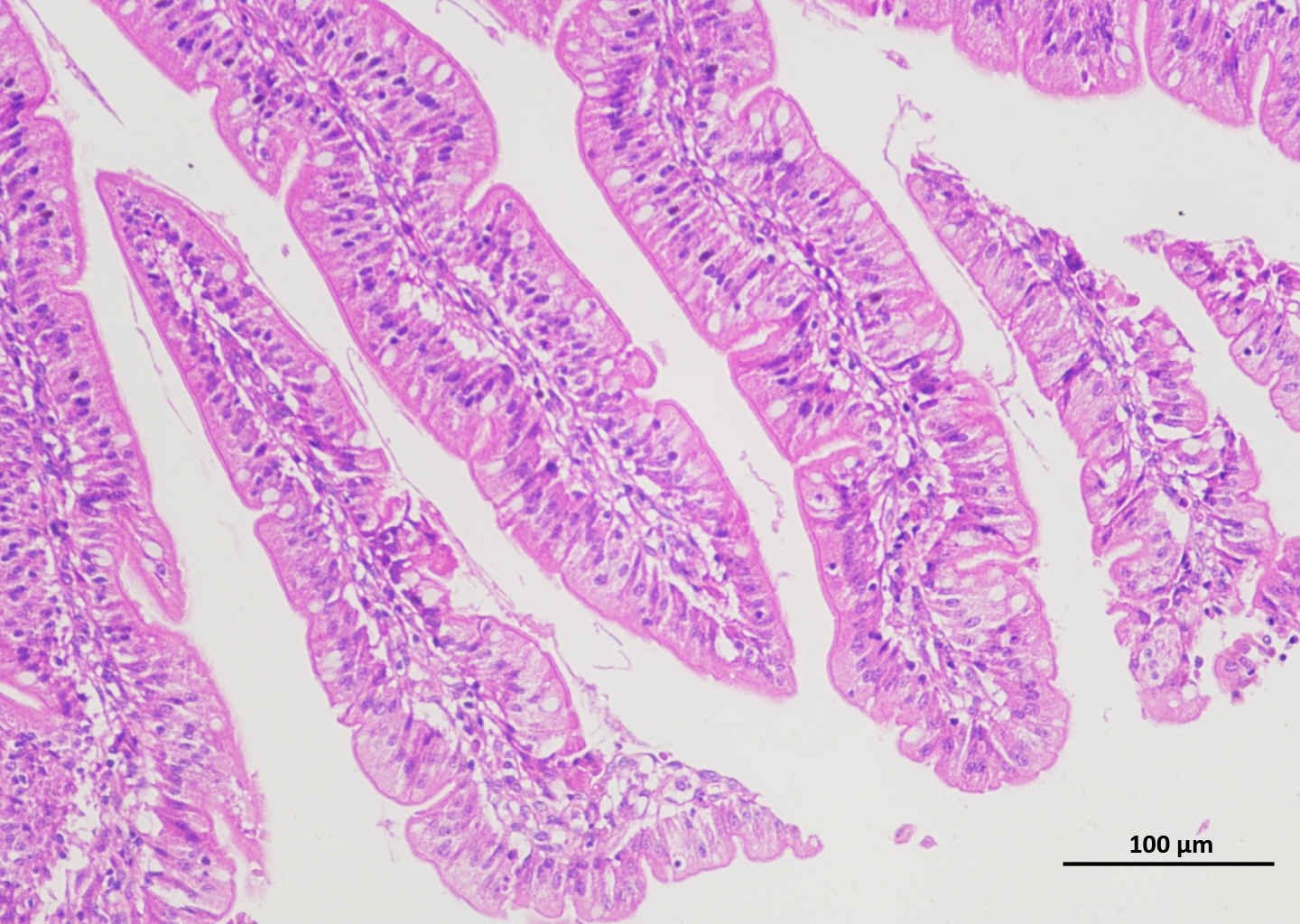 | 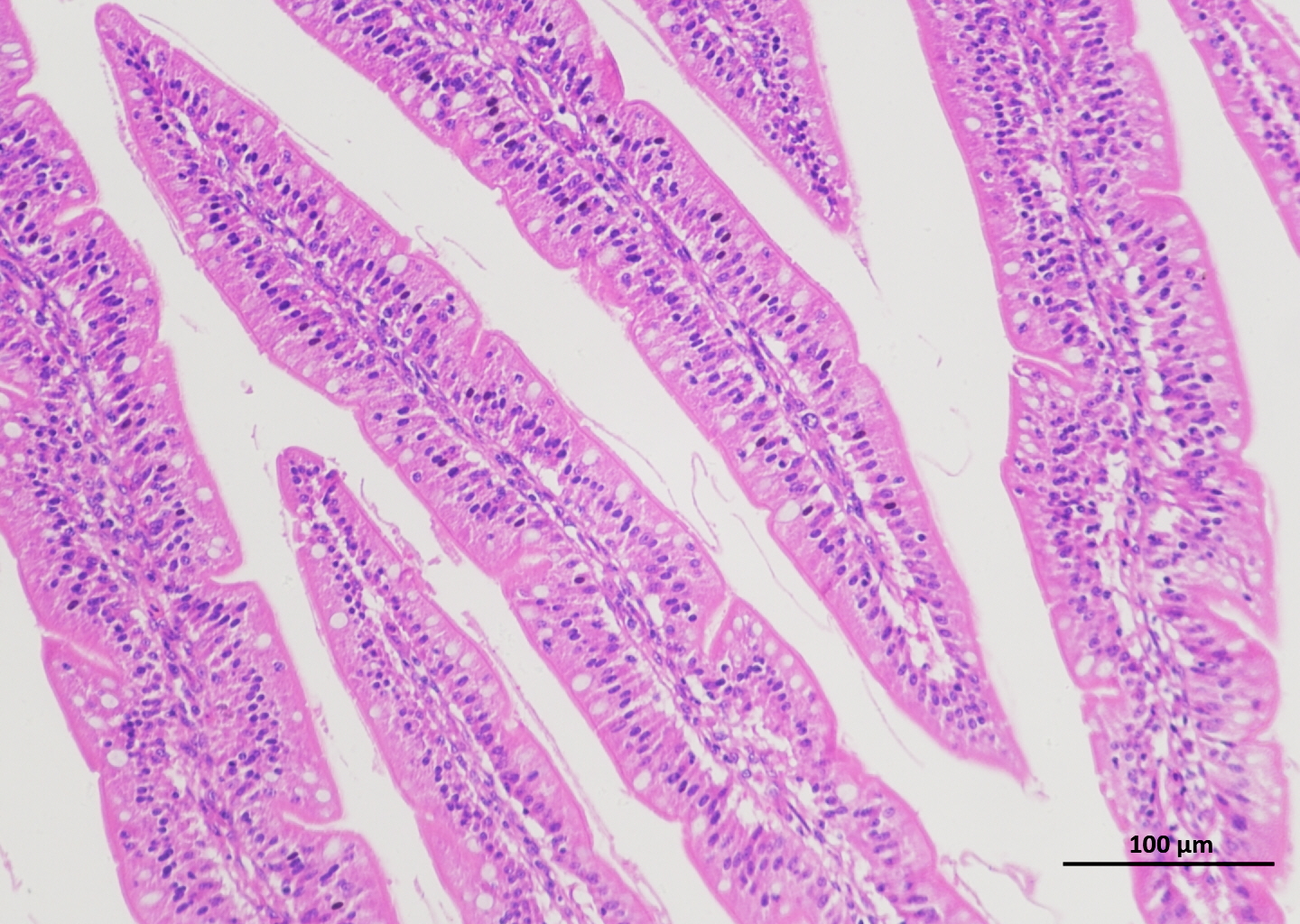 |
| T3-2 | T3-2 |
| The intestinal tissue shows a long length of villi, and the villi epithelium is mainly composed of a single layer of columnar epithelium and a large number of goblet cells; A small amount of intestinal villous epithelial cells show watery degeneration (red arrow), with swollen cells and loose and lightly stained cytoplasm; Occasional shedding of intestinal villous epithelial cells (yellow arrow); The lamina propria is composed of connective tissue and no other obvious abnormalities are observed. | |
| 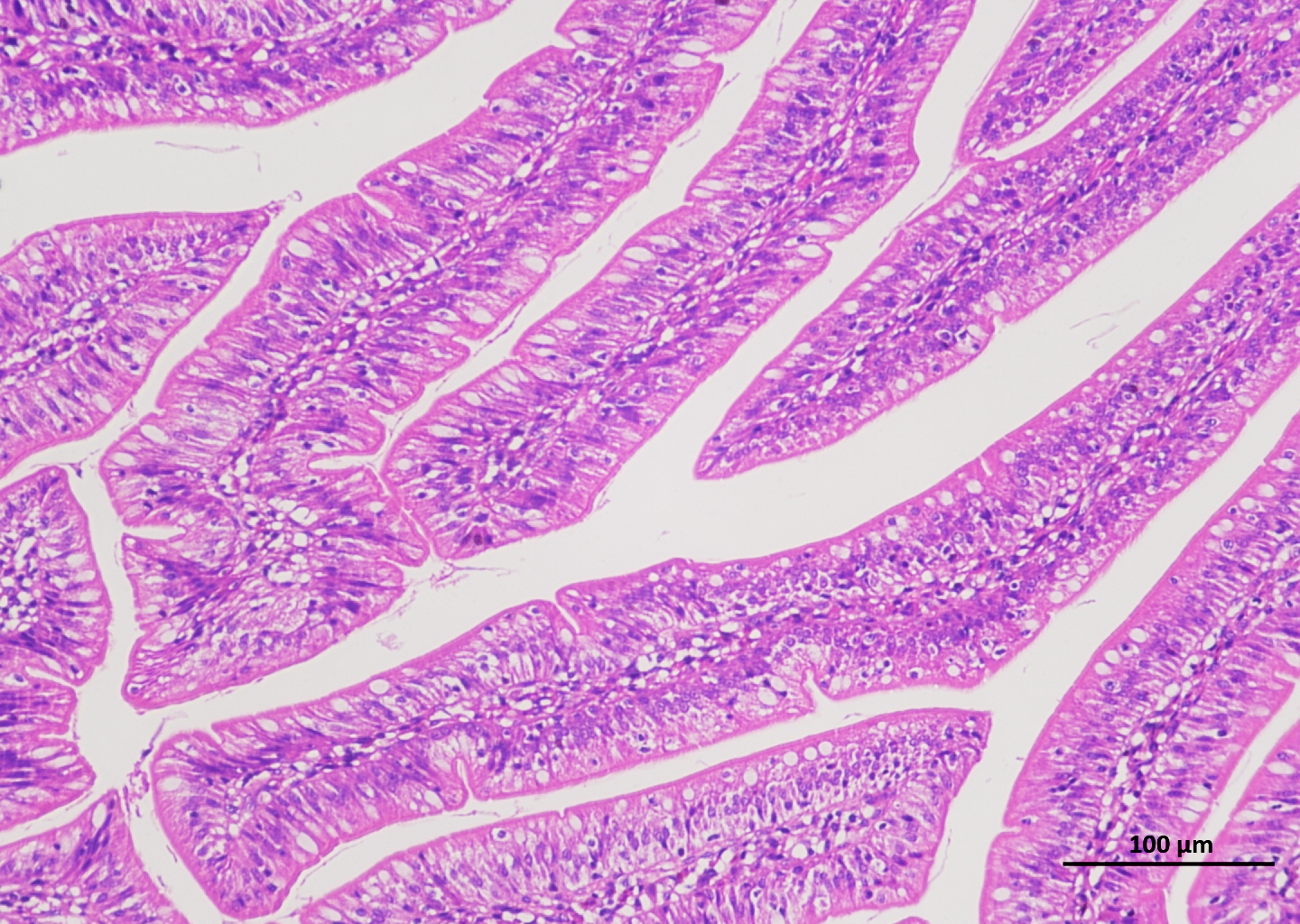 | 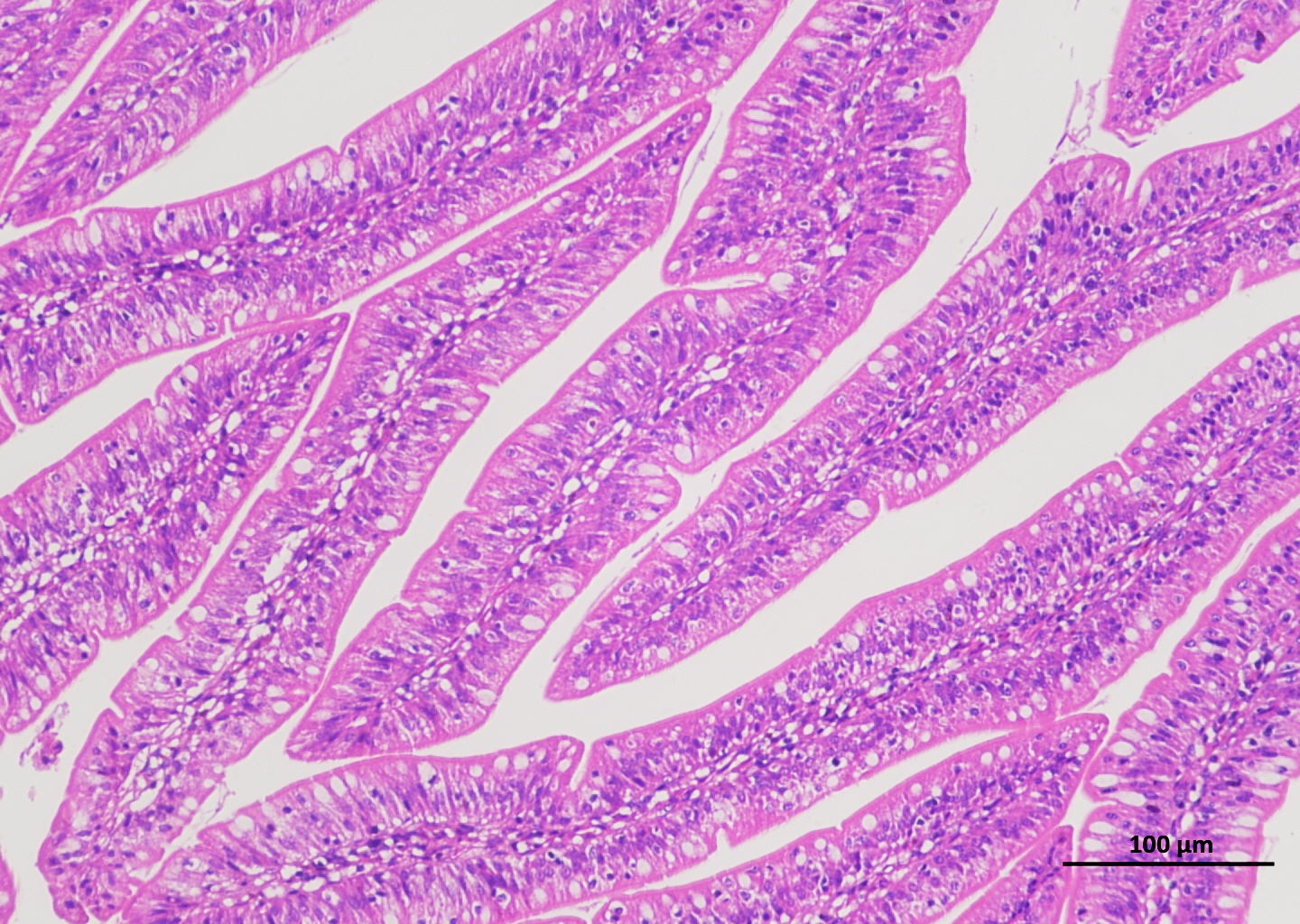 |
| T3-3 | T3-3 |
| The intestinal tissue shows a long length of villi, and the villi epithelium is mainly composed of a single layer of columnar epithelium and a large number of goblet cells; A small amount of intestinal villous epithelial cells show watery degeneration (red arrow), with swollen cells and loose and lightly stained cytoplasm; The lamina propria is composed of connective tissue and no other obvious abnormalities are observed. | |
| 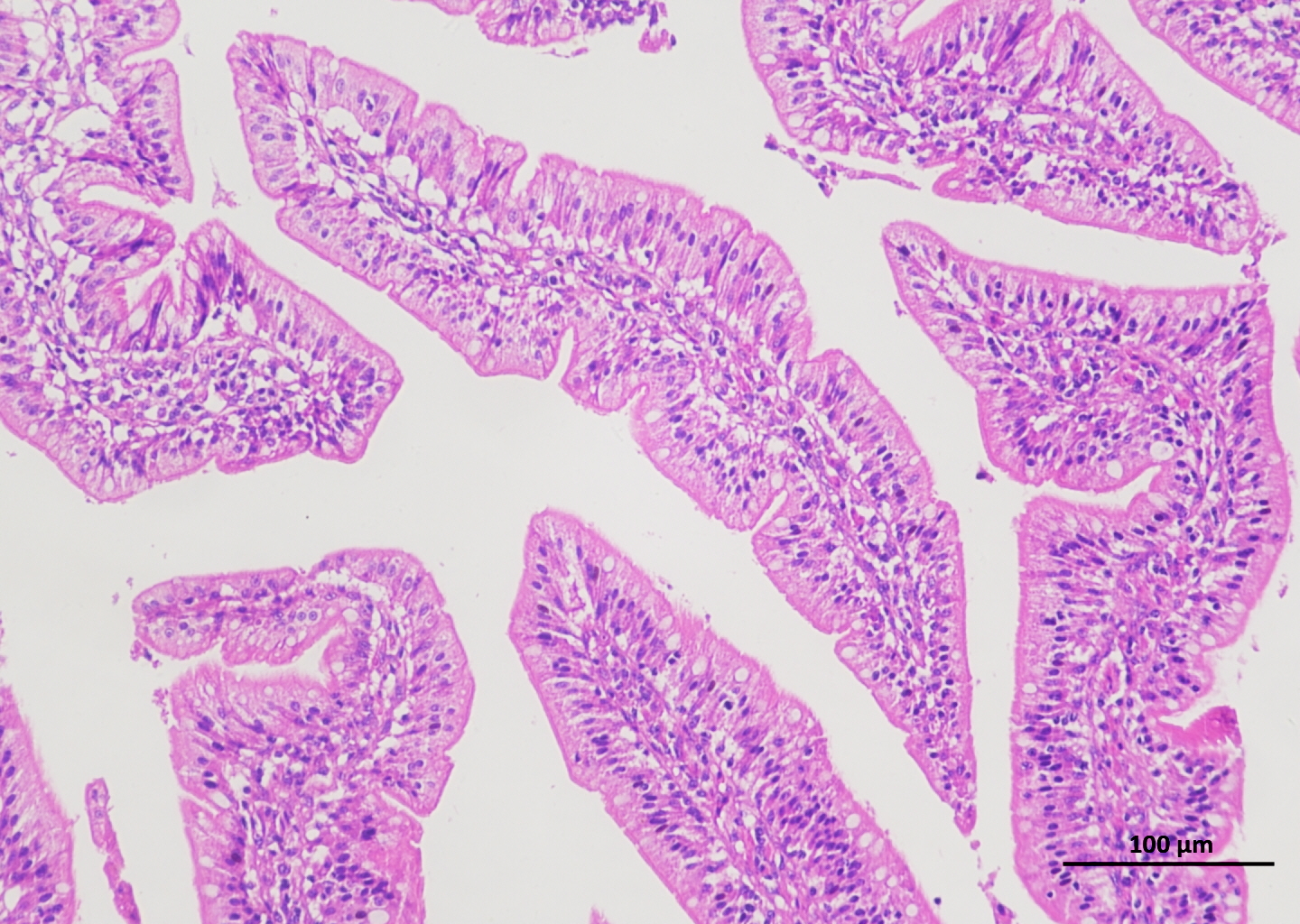 | 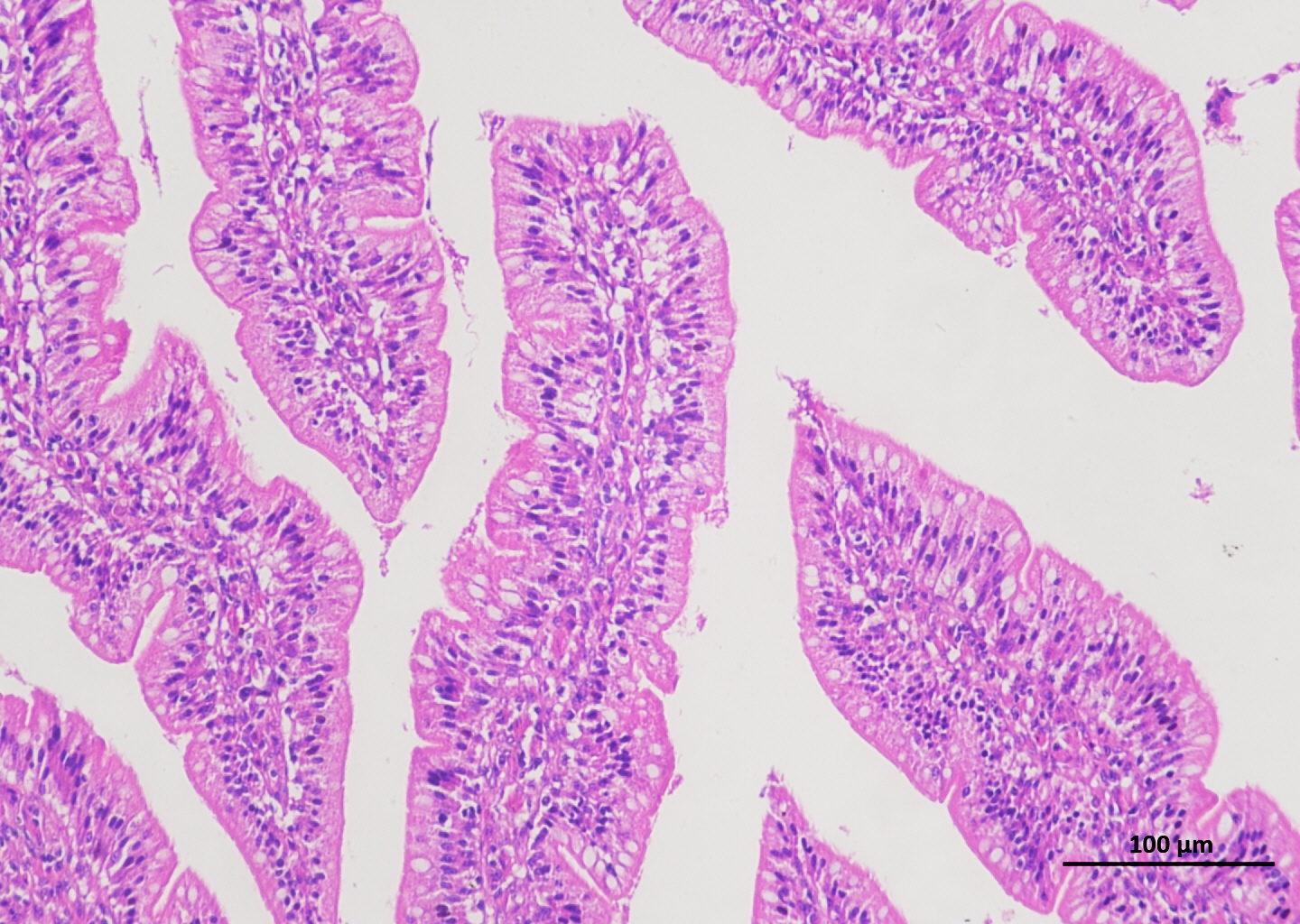 |
| PC-1 | PC-1 |
| The intestinal tissue shows a long length of villi, and the villi epithelium is mainly composed of a single layer of columnar epithelium and a large number of goblet cells; More intestinal villous epithelial cells have watery degeneration (red arrow), swollen cells, and loose and lightly stained cytoplasm; The lamina propria is composed of connective tissue and no other obvious abnormalities are observed. | |
| 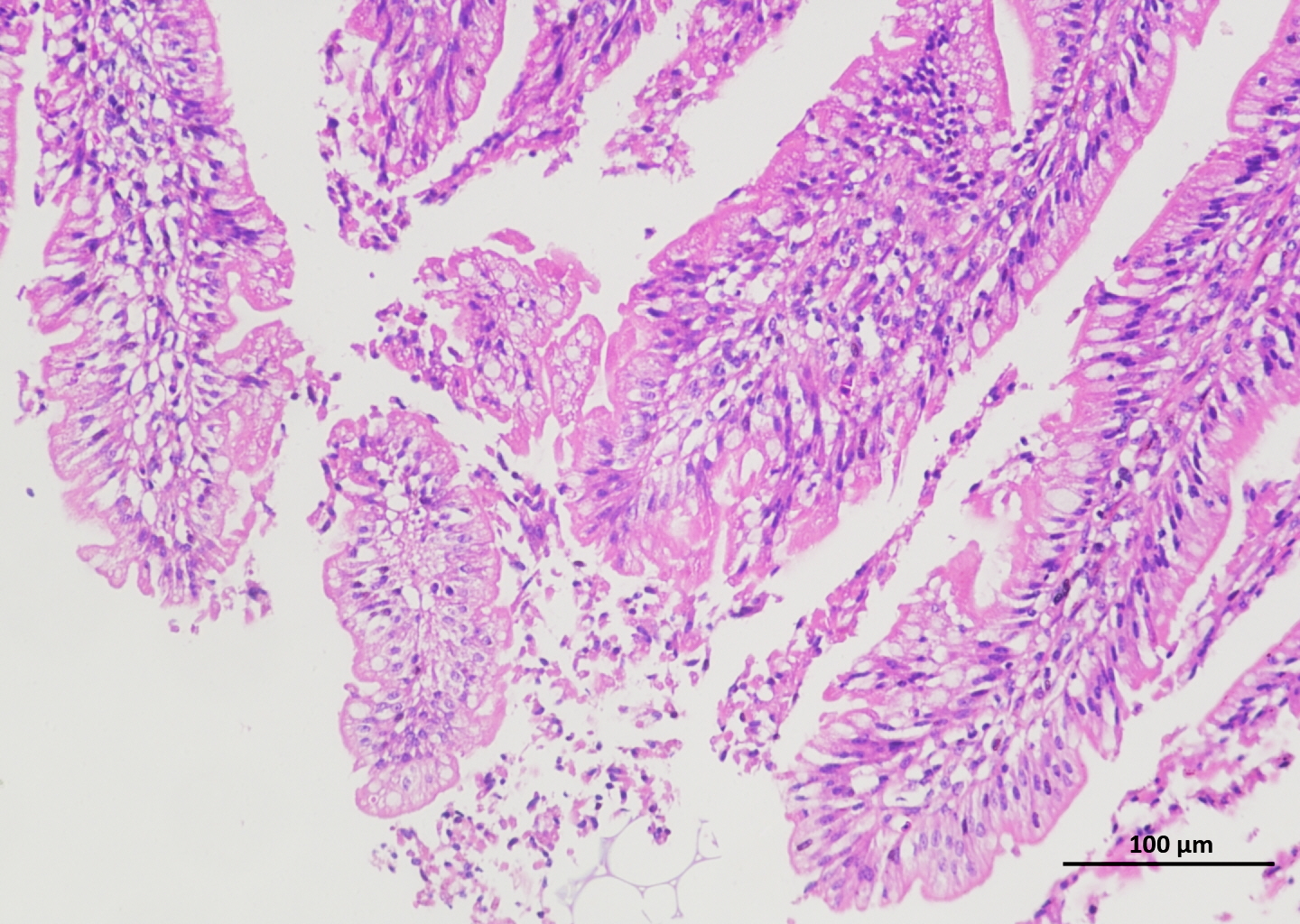 | 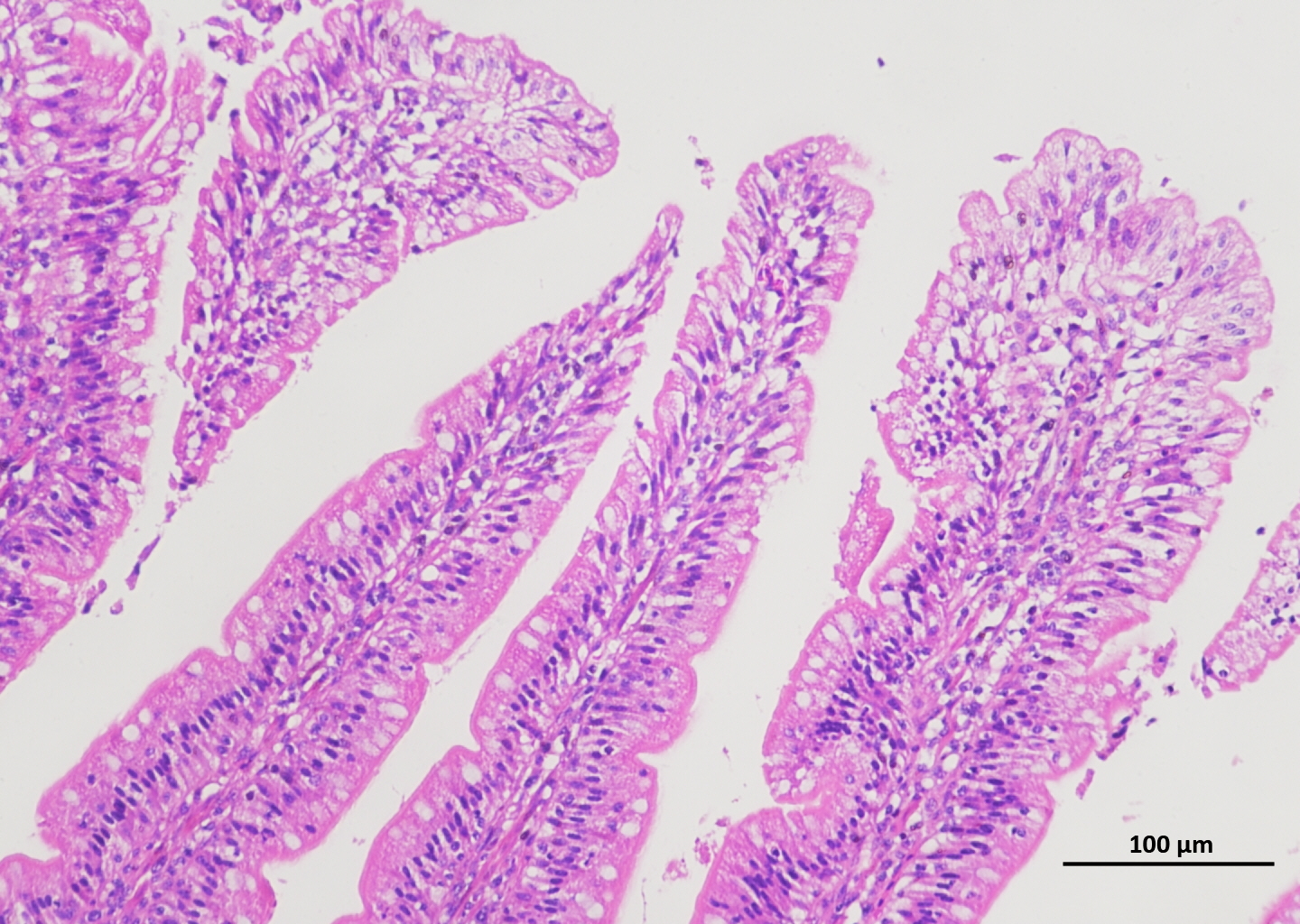 |
| PC-2 | PC-2 |
| The intestinal tissue shows a long length of villi, and the villi epithelium is mainly composed of a single layer of columnar epithelium and a large number of goblet cells; More intestinal villous epithelial cells have watery degeneration (red arrow), swollen cells, and loose and lightly stained cytoplasm; A small amount of intestinal villous epithelial cells shed (yellow arrow); The lamina propria is composed of connective tissue and no other obvious abnormalities are observed. | |
| 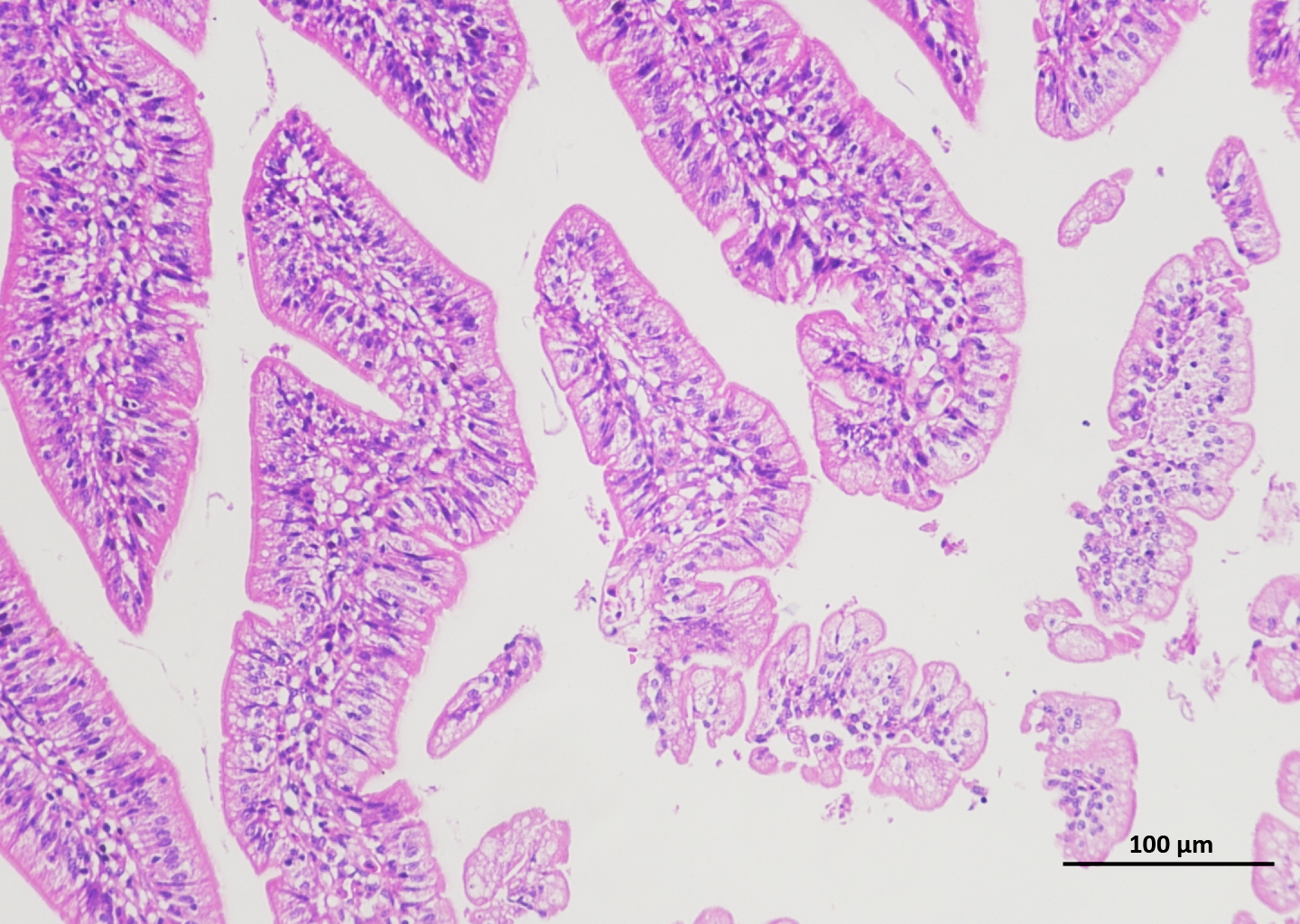 | 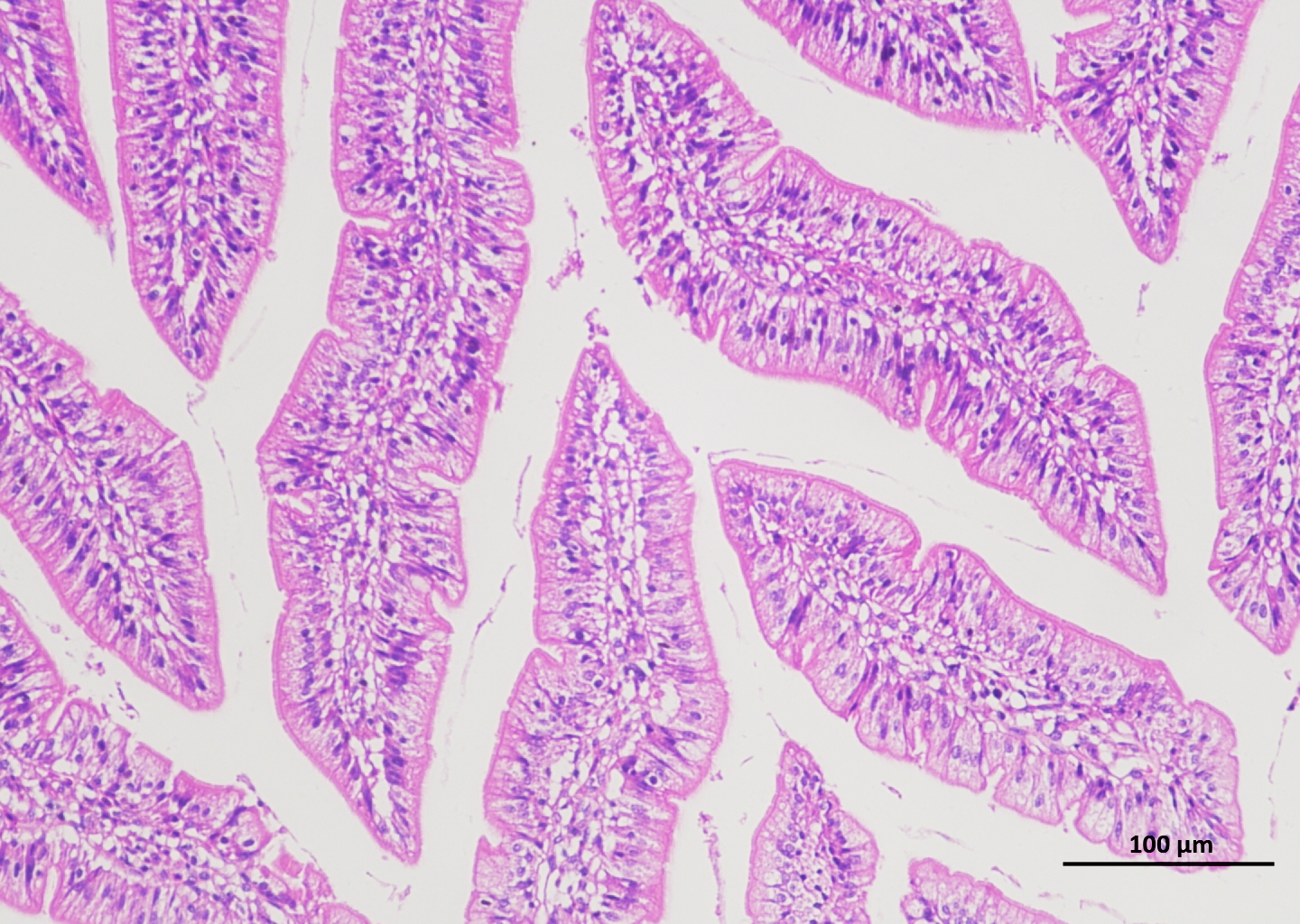 |
| PC-3 | PC-3 |
| The intestinal tissue shows a long length of villi, and the villi epithelium is mainly composed of a single layer of columnar epithelium and a small number of goblet cells; More intestinal villous epithelial cells have watery degeneration (red arrow), swollen cells, and loose and lightly stained cytoplasm; Occasional shedding of villous epithelial cells (yellow arrow); The lamina propria is composed of connective tissue and no other obvious abnormalities are observed. | |
